# Investigating the Metal–TiO_2_ Influence for Highly Selective Photocatalytic Oxidation of Methane to Methanol

**DOI:** 10.1021/acsami.4c02862

**Published:** 2024-07-23

**Authors:** Marcos Augusto R. da Silva, Jéssica C. Gil, Juliana A. Torres, Gelson T. S. T. Silva, José Balena Gabriel Filho, Henrique Fernandes Vieira Victória, Klaus Krambrock, Ivo F. Teixeira, Caue Ribeiro

**Affiliations:** † Nanotechnology National Laboratory for Agriculture (LNNA), Embrapa Instrumentation, São Carlos 13561-206, Brazil; ‡ Department of Chemistry, Federal University of São Carlos (UFSCar), 13565-905 São Carlos, São Paulo, Brazil; § Department of Chemistry, Federal University of Minas Gerais (UFMG), 31270-901 Belo Horizonte, Minas Gerais, Brazil; ∥ Department of Physics, Federal University of Minas Gerais (UFMG), 31270-901 Belo Horizonte, Minas Gerais, Brazil

**Keywords:** photocatalysis, titanium dioxide, methane oxidation, selective oxidation, nickel

## Abstract

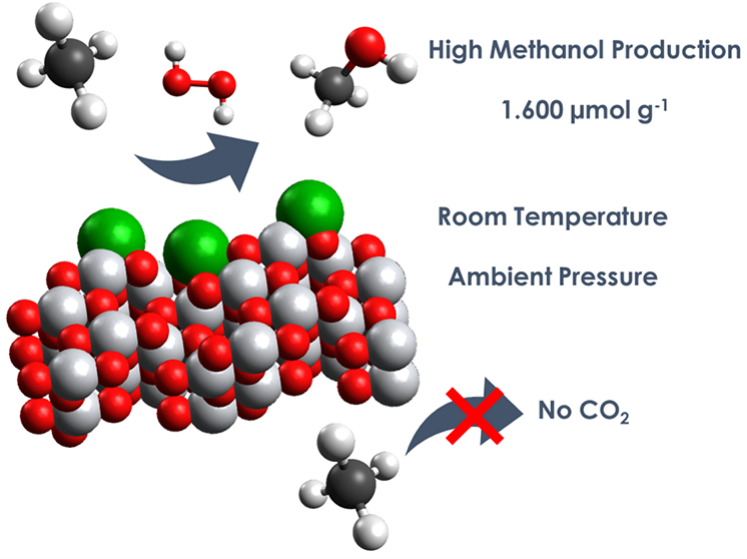

Methane conversion to valuable chemicals is a highly challenging and desirable reaction. Photocatalysis is a clean pathway to drive this chemical reaction, avoiding the high temperature and pressure of the syngas process. Titanium dioxide, being the most used photocatalyst, presents challenges in controlling the oxidation process, which is believed to depend on the metal sites on its surface that function as heterojunctions. Herein, we supported different metals on TiO_2_ and evaluated their activity in methane photooxidation reactions. We showed that Ni–TiO_2_ is the best photocatalyst for selective methane conversion, producing impressively high amounts of methanol (1.600 μmol·g^–1^) using H_2_O_2_ as an oxidant, with minimal CO_2_ evolution. This performance is attributed to the high efficiency of nickel species to produce hydroxyl radicals and enhance H_2_O_2_ utilization as well as to induce carrier traps (Ti^3+^ and SETOVs sites) on TiO_2_, which are crucial for C–H activation. This study sheds light on the role of catalyst structure in the proper control of CH_4_ photoconversion.

## Introduction

Methane, constituting 70–90% of natural gas, has emerged as a compelling energy source and a viable alternative to nonrenewable petroleum resources, particularly with the recent discoveries of natural gas hydrate reserves.^1−3^ Furthermore, methane is a crucial raw material for fuel and chemical production.^4,5^ However, extracting it from remote locations and transporting it over long distances pose significant challenges.^6^ Moreover, methane is a potent greenhouse gas, with a global warming potential 25 times higher than that of carbon dioxide (CO_2_).^6,7^ Consequently, there is a pressing need to explore methods for converting methane into other valuable chemicals. Significantly, the conversion of methane to methanol offers an avenue to produce liquid fuel for energy generation or to serve as a foundational component for high-value chemicals.^8^ The current industrial process for converting methane to methanol follows an indirect route, involving syngas formation at high temperatures (>700 °C) and subsequent alcohol conversion under high pressures (>10 atm).^9−11^ While effective, this method is energy-intensive and costly. Therefore, there is a critical need to develop direct pathways for converting methane into methanol, particularly under mild reaction conditions.^12−14^

Photocatalysis offers a potential pathway for methane activation through photons, instead of high temperatures, to drive chemical reactions under mild conditions (ambient pressure and temperature).^15−17^ Photocatalytic CH_4_ activation can proceed via direct or indirect routes: indirect processes utilize active radicals (such as hydroxyl) to abstract hydrogen from CH_4_, while direct activation occurs through surface defects (oxygen vacancies or O^–^).^3,15^ The photocatalyst design to improve such processes is highly desirable. As the most prominent and applied photocatalyst, titanium dioxide (TiO_2_) is one of the best candidates to be a suitable catalyst for methane conversion into valuable chemicals.^18−21^ Although studies have demonstrated TiO_2_ efficiency in producing oxygenated products, several strategies have been employed to enhance the efficacy of this photocatalyst for methane oxidation. These strategies include the formation of heterojunctions between different semiconductors or various TiO_2_ phases, the generation of surface defects to enhance CH_4_ activation, and the stabilization of metal species that can serve as cocatalysts.^20−23^ The metal introduction appears to produce highly active materials by improving charge separation, creating surface defects, and generating selective oxygen radicals. For instance, iron species onto TiO_2_ improve the production of methanol using H_2_O_2_ as oxidant.^21^ However, considering that other transition metals are reported for reactions that can promote or compete with CH_4_ oxidation, we hypothesize that they can interfere with the byproduct selectivity. A deeper investigation of other transition metals over TiO_2_, using H_2_O_2_ as an oxidant, can reveal how to control this reaction leading to specific oxygenates.

Considering this gap, we synthesized different metal-supported TiO_2_ photocatalysts using an impregnation method and investigated their activity for methane photooxidation reactions to liquid oxygenates using H_2_O_2_ as an oxidant. As expected, different activities were observed, as well as different products, which are related to different reaction mechanisms. Most surprisingly, among noble and non-noble metals, Ni–TiO_2_ exhibits the highest methanol production (1.600 μmol·g^–1^), which is correlated to the high efficiency of nickel species to produce hydroxyl radicals and the high amount of carrier traps in TiO_2_ to activate CH_4_ molecules. To the best of our knowledge, our result is one of the highest methanol yields from methane under ambient conditions (25 °C and 1 bar).

## Results and Discussion

### Metal–TiO_2_ Photocatalysts Synthesis

**Figure 1:**
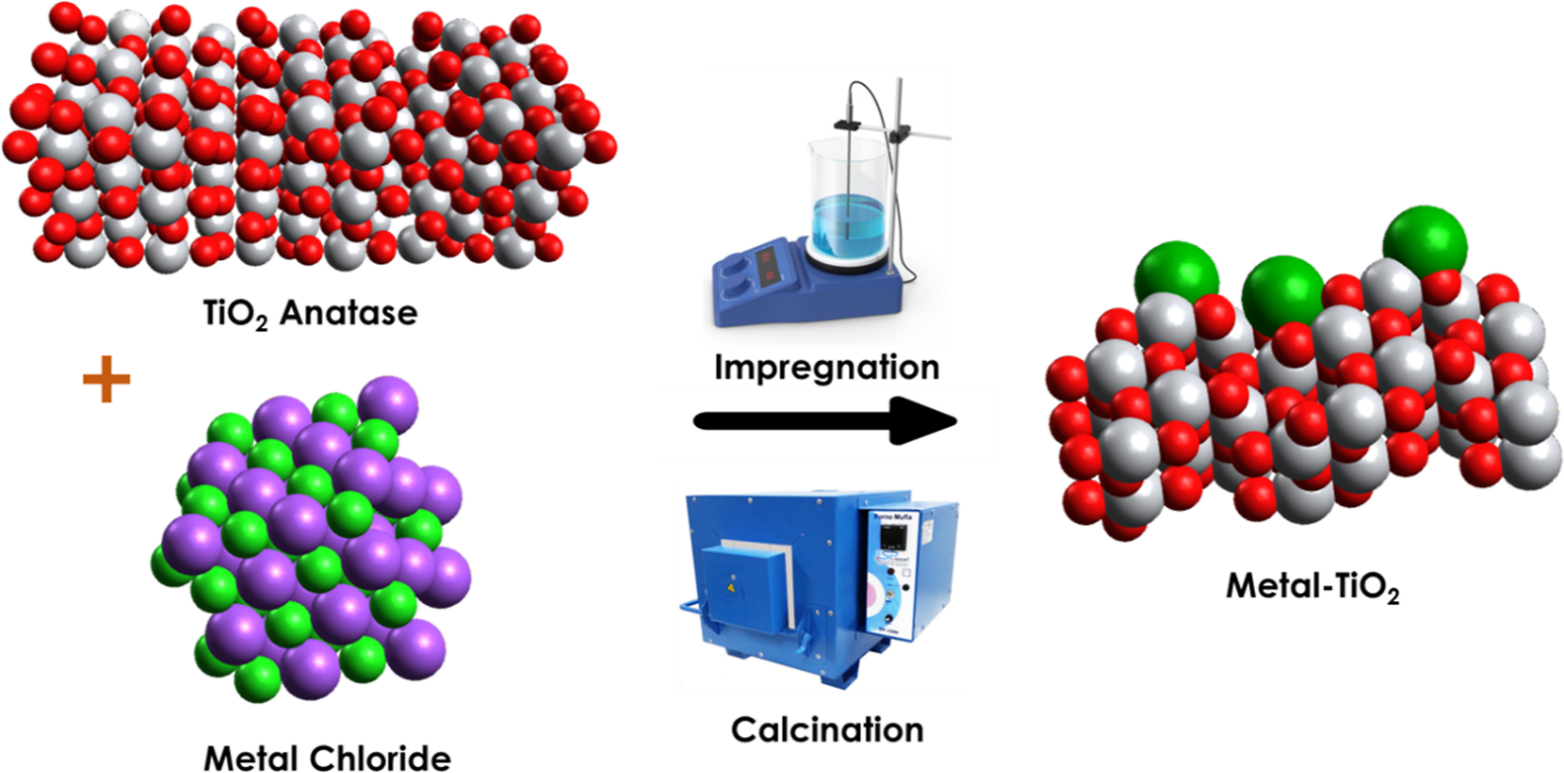
Synthetic procedure to fabricate metal–TiO_2_ photocatalysts. Colors: oxygen (red), chlorine (violet), titanium (gray), and metal species (green).

The catalysts were prepared using a wet impregnation method, wherein solutions of metal chlorides (Co, Cu, Ni, Pd, and Ag) were stirred with a commercial TiO_2_ anatase. Subsequently, the solvent was evaporated, and the samples were calcined at 400 °C for 4 h (Figure 1). Figure S1 depicts the powder X-ray diffraction (XRD) patterns of the as-prepared samples. It is observed that all catalysts exhibit peaks associated with the tetragonal anatase phase of TiO_2_ (JCPDS 84-1285), indicating the preservation of the material’s structure even after metal impregnation. Additionally, no diffraction patterns corresponding to the metal particles were identified, even for noble metals (Pd and Ag), indicating their high dispersion on the TiO_2_ surface. The average crystallite size for TiO_2_ was calculated from the (101) plane, the most intense peak, and ranged from 22.97 to 28.21 nm (see Table S1).

The quantities of metallic species in the photocatalysts were assessed by using atomic absorption spectroscopy and inductively coupled plasma optical emission spectroscopy. As shown in Table S2, the metal loadings closely matched the expected values, highlighting the efficiency of this method. The specific surface areas, calculated using the Brunauer–Emmett–Teller (BET) method, were determined for pristine TiO_2_ and TiO_2_ loaded with metals (Table S2). The impregnation of metals and subsequent calcination did not significantly impact the S_(BET)_ compared to pristine TiO_2_. These areas do not correspond to the fully available surfaces of metallic species but rather to the surfaces of agglomerated particles. Moreover, the optical properties of the photocatalysts were examined through diffuse reflectance UV–vis spectra (Figure S2), revealing the expected UV absorption characteristics of titanium dioxide materials.

### Methane Photocatalytic Oxidation Tests

**Figure 2:**
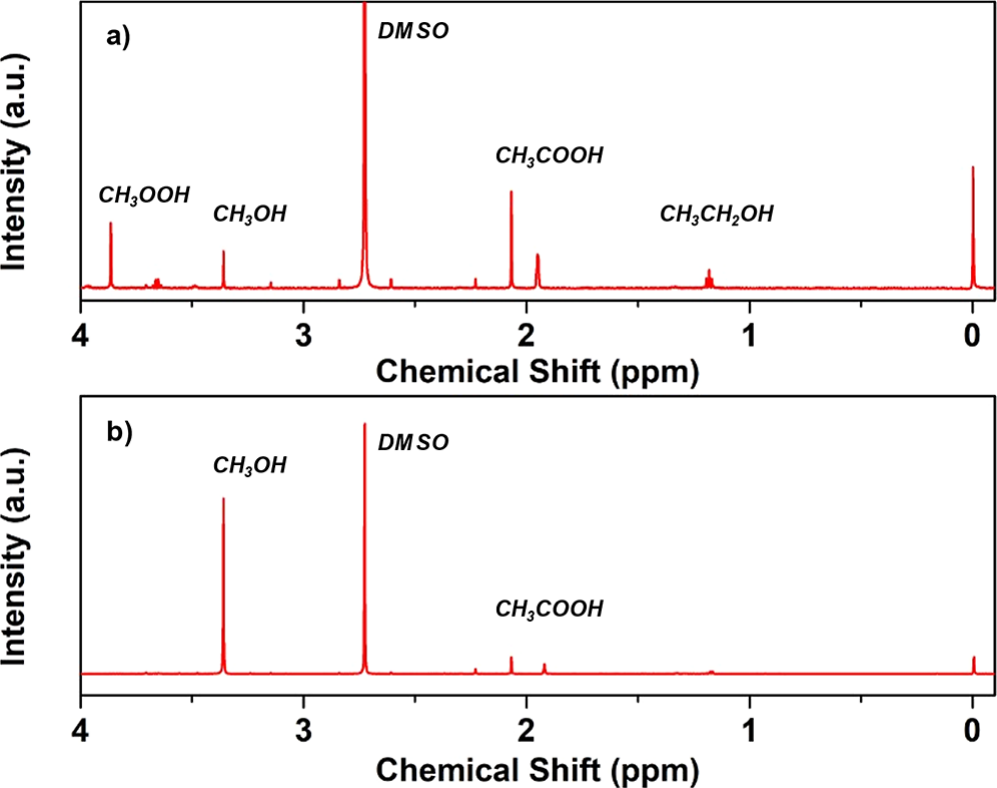
^1^H NMR spectra of reaction products from Ag–TiO_2_ (a) and Ni–TiO_2_ (b).

**Figure 3:**
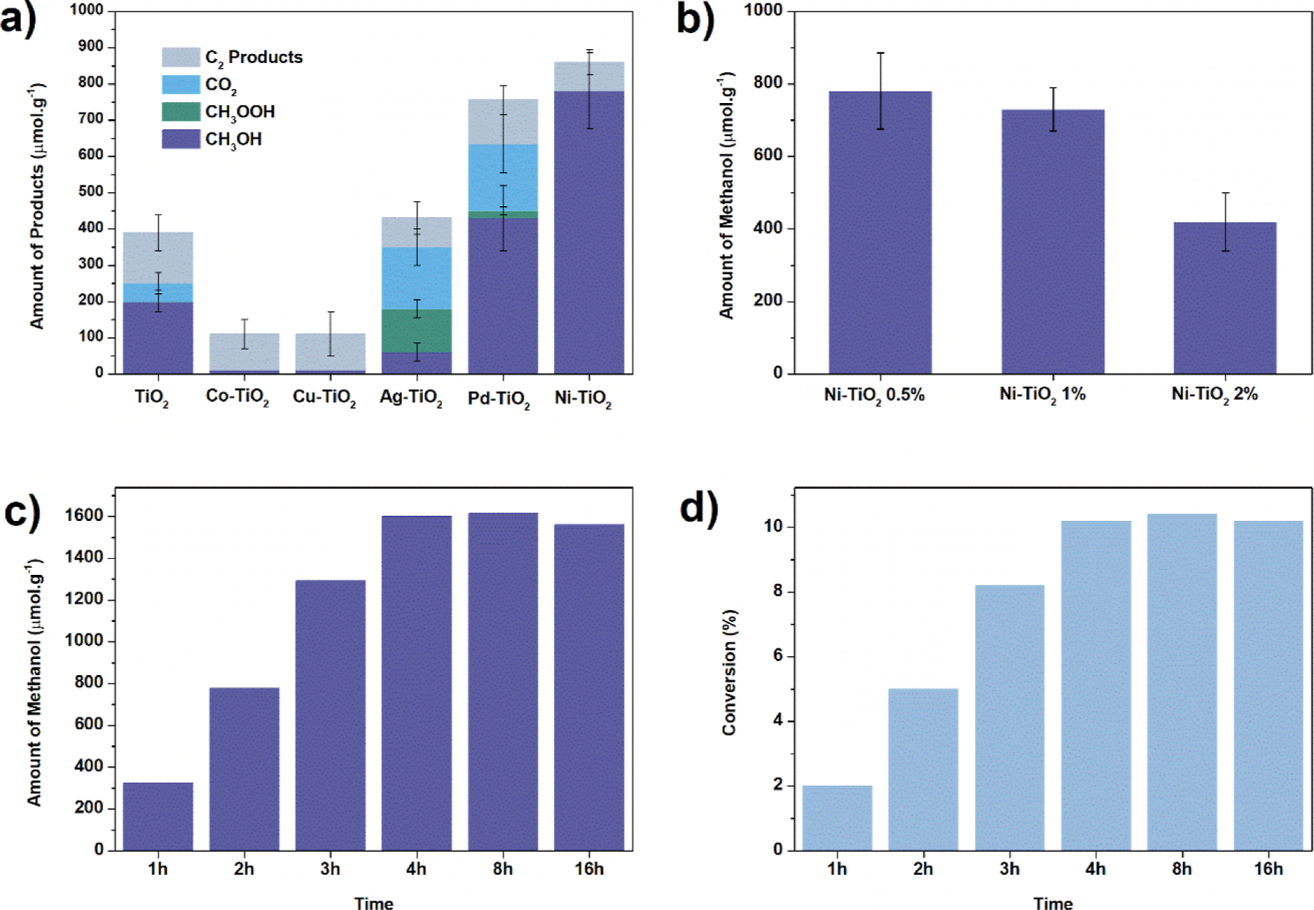
(a) Product distribution for a series of TiO_2_ samples modified with metals (2 h of reaction); (b) methanol yield with different amounts of Ni (0.5, 1, and 2%) after 2 h of reaction; (c) evolution of methanol concentration along reaction time; (d) methane conversion into oxygenates (see Supporting Information for details). Reaction conditions: 100 mg of photocatalyst, 2 mM H_2_O_2_ in 100 mL of H_2_O, and CH_4_ (99.9%) at room temperature and atmospheric pressure.

The synthesized materials were employed in photocatalytic methane oxidation reactions conducted in a quartz tube featuring a saturated CH_4_ atmosphere with hydrogen peroxide (H_2_O_2_) as the oxidant under UV irradiation and ambient conditions (1 bar, 25 °C) (for more details, see Supporting Information). As depicted in Figure 2a, Ni–TiO_2_ demonstrated highly selective methanol production within 2 h, contrasting with noble-metal-supported materials and pristine TiO_2_, which exhibited a less selective distribution (Figure 3a). Specifically, Pd and Ag–TiO_2_ generated substantial amounts of carbon dioxide, indicating the overoxidation of methane products by these photocatalysts. Notably, in the noble-metal tests, the nuclear magnetic resonance (NMR) spectra revealed the presence of CH_3_OOH (Figure 2a), a common intermediate in H_2_O_2_-mediated methane oxidation reactions.^24−26^ However, this compound was not identified in tests catalyzed by Ni–TiO_2_ (Figure 2b). Hence, it is evident that the mechanism for Pd and Ag–TiO_2_ materials differs from that of Ni–TiO_2_, possibly involving the formation of •OOH radicals. Ni–TiO_2_ stands out as the more active photocatalyst for generating significant quantities of methanol while minimizing the level of CO_2_ production. Given the high activity observed for Ni–TiO_2_, this material underwent further investigation with varying nickel loadings.

**Figure 4:**
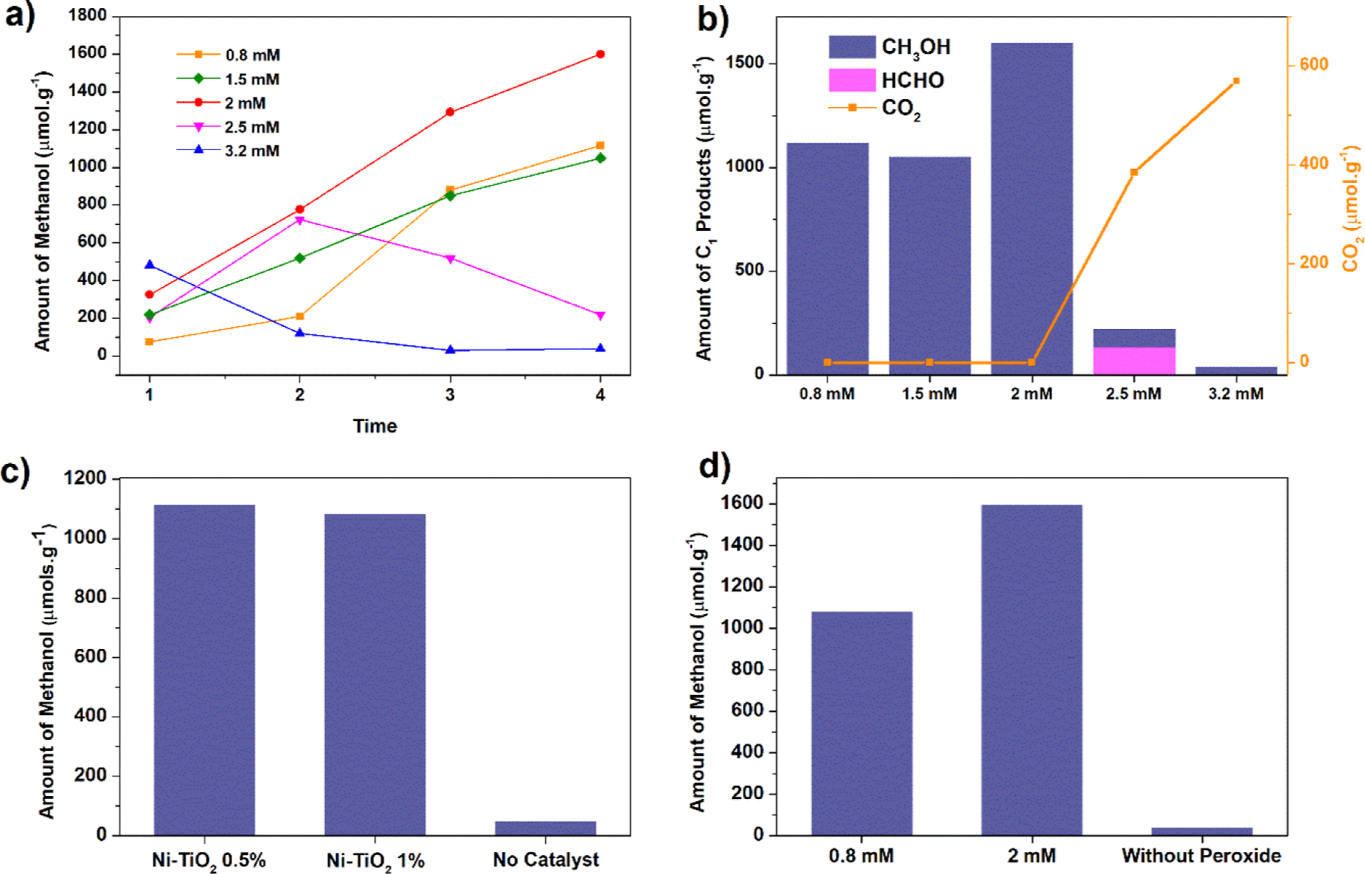
(a) Methanol yield with different H_2_O_2_ concentrations (1–4 h of reaction); (b) amount of C_1_ products for different H_2_O_2_ concentrations (4 h of reaction); (c) comparison of methanol yield of Ni–TiO_2_ materials, with reaction carried out without a catalyst (4 h of reaction and 0.8 mM H_2_O_2_); (d) comparison of methanol yield with different H_2_O_2_ concentrations and in the absence of peroxide (Ni–TiO_2_ 0.5%, 4 h of reaction); reaction conditions: 100 mg of photocatalyst, 2 mM H_2_O_2_ in 100 mL of H_2_O, and CH_4_ (99.9%) operated at room temperature and atmospheric pressure.

Ni–TiO_2_ photocatalysts with different nickel concentrations were produced by the same method as described earlier. An exploration of metal loading for Ni materials (Figure 3b) reveals that a high dispersion of metallic sites enhances methanol generation, and optimal production is observed for Ni–TiO_2_ 0.5%. After a 4 h reaction period, we achieved the highest production of oxygenated liquids, with a yield of 1600 μmol·g^–1^ of methanol and 170 μmol·g^–1^ of C_2_ products (ethanol and acetic acid). At this juncture, the total methane conversion into oxygenates reaches 10% (Figure 3d). Beyond 4 h, the production plateaus and remains unchanged (Figure 3c). Intriguingly, even after 16 h, the methanol production does not change, while CO_2_ evolution slightly increases (Figure S3). It indicates that, under our conditions, the system approaches an equilibrium state between methanol and CO_2_ production. Furthermore, Figure 4a illustrates that 2 mM was the optimal concentration of H_2_O_2_ for achieving high methanol quantities, indicating that excessive amounts of peroxide could potentially result in the degradation of oxygenates. Interestingly, when we increased the H_2_O_2_ concentration to 2.5 mM, we observed the formation of formaldehyde (HCHO) as a byproduct, in addition to CO_2_ (Figure 4b). This suggests that formaldehyde molecules are generated under highly oxidative conditions and rapidly degrade into CO_2_. This hypothesis is supported by our findings when using 3.2 mM H_2_O_2_, where we only observed carbon dioxide as a byproduct after 4 h, along with minimal amounts of methanol.

The hypothesis that hydrogen peroxide itself can oxidize methane could not be excluded since H_2_O_2_ breaks into hydroxyl radicals under UV radiation. Tests without a catalyst were performed (Figure 4c), wherein only 50 μmol·g^–1^ of methanol was quantified with CO_2_ as the primary product (130 μmol·g^–1^), indicating that Ni species are crucial for promoting methanol generation. The significance of hydrogen peroxide in the reaction is evident as a substantial decrease occurs when the oxidant is not present (Figure 4d). The absence of product under dark conditions emphasizes the necessity of irradiation for methanol production. Furthermore, the potential production of methanol from other carbon sources in the medium was ruled out as no product was detected in the absence of CH_4_ (Figure S4).

### Ni–TiO_2_ Characterization

**Figure 5:**
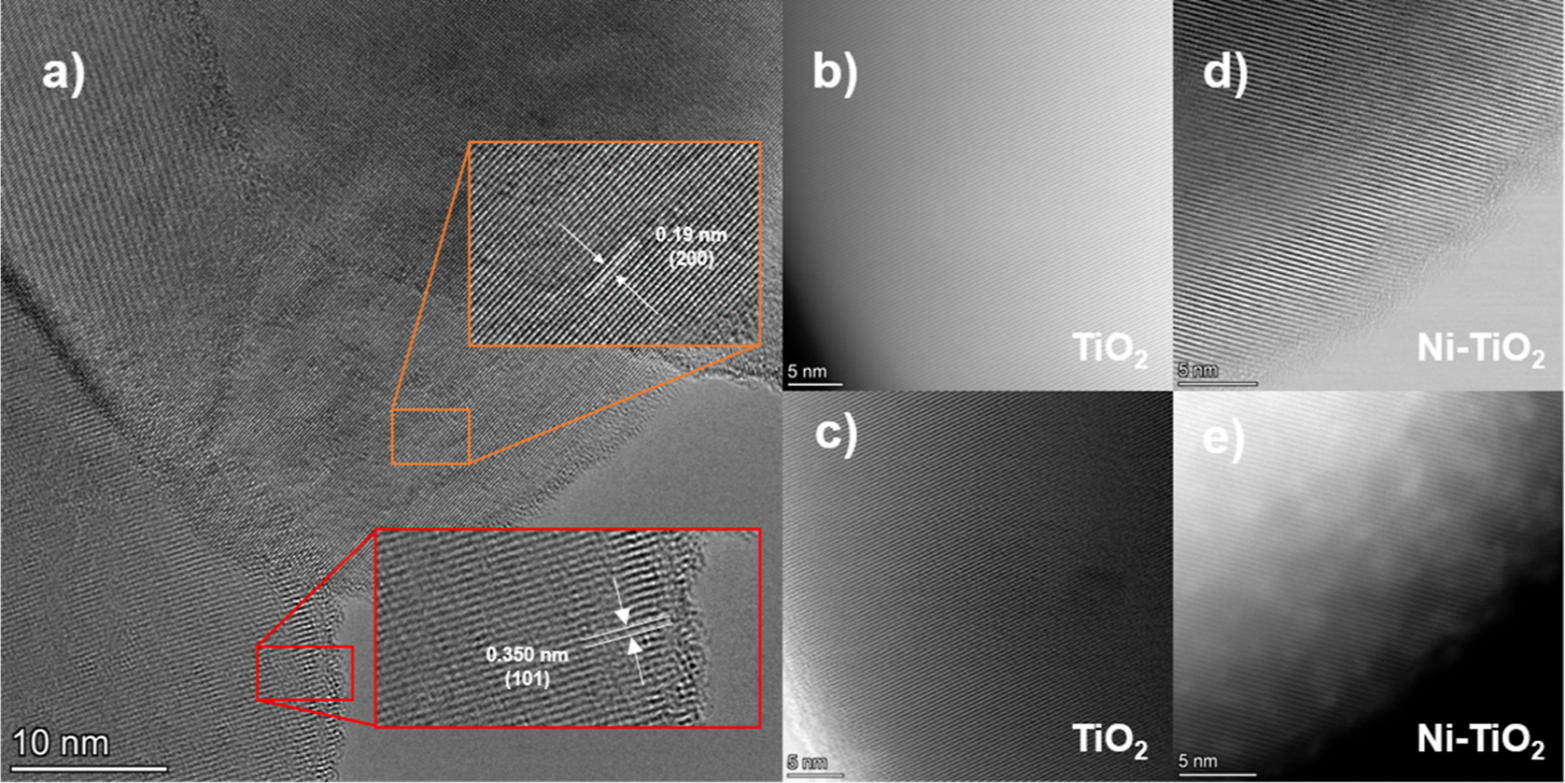
(a) HRTEM and (b,c) HAADF-STEM images of TiO_2_ and (d) HRTEM and HAADF-STEM images of Ni–TiO_2_ 0.5%.

SEM micrographs of TiO_2_ and Ni–TiO_2_ 0.5 and 2% are shown in Figure S5. The materials presented similar morphologies, formed by particle aggregates and with no apparent change in size or shape after nickel impregnation. However, it is possible to observe roughness on the Ni–TiO_2_ 2% surface (Figure S5c), possibly caused by the higher amount of nickel species in this sample. The morphologies of Ni–TiO_2_ of 0.5% and pristine TiO_2_ were also determined by transmission electron microscopy (TEM) (Figure S6). HRTEM images (Figure 5a) show lattice fringes of 0.350 and 0.19 nm, which are related to the (101) and (200) planes of TiO_2_, respectively.^27,28^ It is interesting to note a lattice distortion, which is observed in SAED patterns (Figure S7), which indicates possible nickel doping. To verify the dispersion of nickel sites, high-angle annular dark field scanning transmission electron microscopy (HAADF-STEM) images were acquired (Figure 5b–e). A notable distinction between the TiO_2_ and Ni–TiO_2_ images is evident, whereby we can observe bright dots on TiO_2_ support (Figure 5e), related to nickel clusters, which are noticed on the pure material (Figure 5b,c).

**Figure 6:**
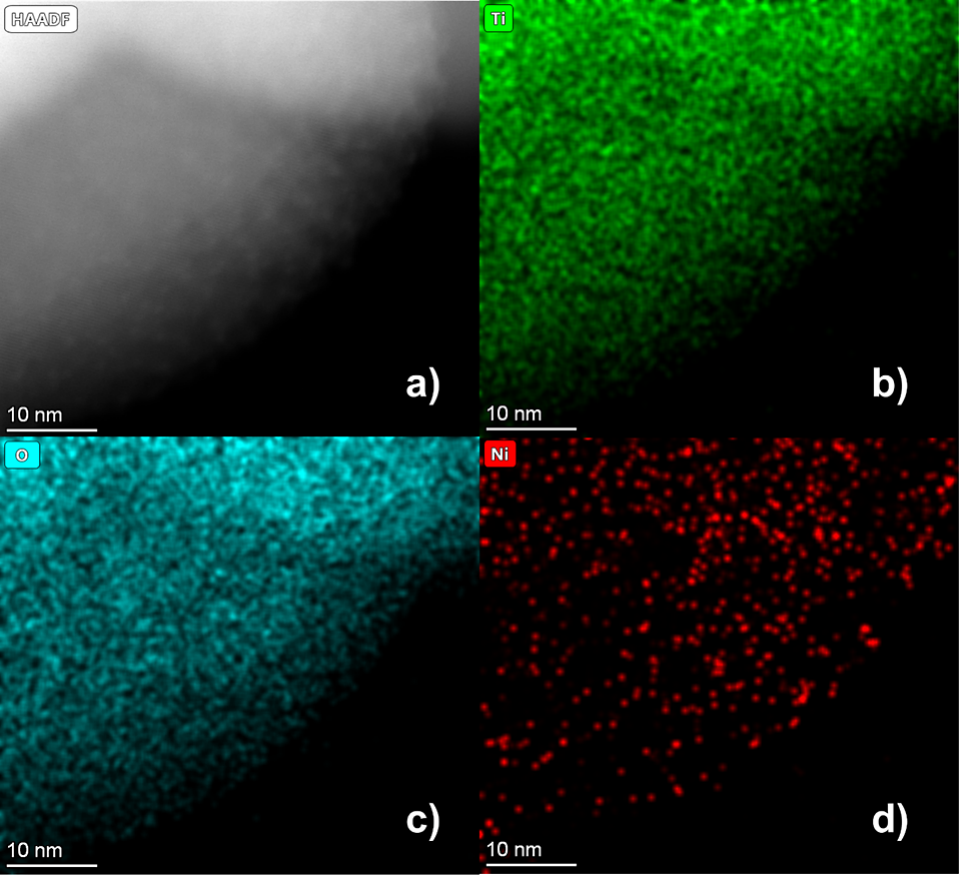
EDS mapping obtained from HAADF-STEM images showing Ti, O, and Ni atoms on the Ni–TiO_2_ material.

**Figure 7:**
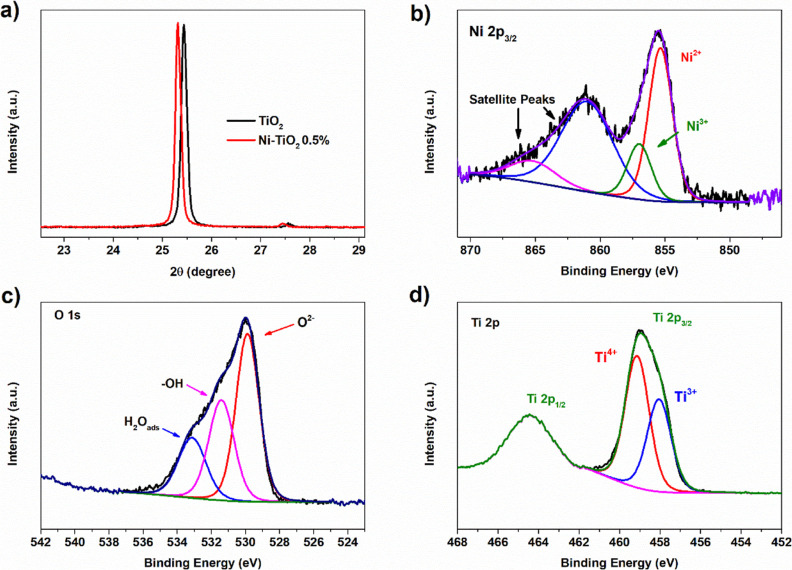
(a) XRD patterns of TiO_2_ and Ni–TiO_2_ showing peak shift related to nickel doping; high-resolution XPS spectra of (b) Ni 2p, (c) O 1s, and (d) Ti 2p of Ni–TiO_2_ 0.5%.

The high dispersibility of Ni was further confirmed by EDS mapping (Figure 6). The images reveal nickel sites ranging in size from 0.3 to 1.4 nm, which can be categorized as Ni clusters. At this scale, it is expected that some nickel atoms are introduced in the TiO_2_ lattice as dopants. Besides the SAED pattern, clear indications of lattice distortion are evident in the XRD diffractograms of TiO_2_ and Ni–TiO_2_ (Figure 7a), which exhibit shifts toward smaller angles upon the introduction of nickel, suggesting an increased interplanar distance.

Survey spectra of Ni–TiO_2_ 0.5% and Ni–TiO_2_ 2% samples are shown in Figure S8 and reveal the presence of the expected elements: Ti and O, as well as Ni. The surface species on Ni–TiO_2_ was investigated with high-resolution X-ray photoelectron spectroscopy (XPS). Figure 7b shows the high-resolution XPS for Ni 2p_3/2_ in Ni–TiO_2_; the deconvolution of the peaks indicates the presence of Ni^2+^ species and Ni^3+^ species, suggesting that nickel clusters can be identified as NiO_*x*_.^29,30^ This finding is consistent with prior research utilizing the same impregnation method, which also identified the presence of NiO_*x*_ clusters.^31^ Also, Ni(0) signals are absent in the XPS spectra, excluding the presence of nanoparticles in the sample.^32^ The high-resolution spectrum of O 1s (Figure 7c) can be deconvoluted into three peaks at 529.8, 531.4, and 533.1 eV, which are attributed to lattice oxygen (O^2–^), hydroxyl groups (−OH) or oxygen vacancies (O_v_), and surface-adsorbed water (H_2_O).^31,33^ Also, in the Ti 2p spectra, we identify the presence of Ti^3+^ and Ti^4+^ sites (Figure 7d), suggesting that Ni dopants introduce defects in the structure.^34,35^

The Raman spectrum of TiO_2_ (Figure S9a) shows typical signals related to the anatase TiO_2_ phase at 143.6, 197.4, 395, 513.6, and 636.5 cm^–1^, assigned as Eg, Eg, B1, A1g + B1g, and Eg modes, respectively.^36,37^ The introduction of Ni species into the support does not add any peaks in the Raman spectrum. This result corroborates XRD analyses, which exhibit the same diffraction pattern for all samples. However, when NiO_*x*_ clusters are loaded into TiO_2_, a small shift occurs in the spectrum (Figure S9b), which could be derived from the lattice distortion caused by Ni doping. Figure S10 shows photoluminescence (PL) spectra of pure TiO_2_, Ni–TiO_2_ 0.5%, and Ni–TiO_2_ 2%. Typical bands related to TiO_2_ are exhibited for all samples, especially in the region of 400–450 nm, which is assigned to the recombination of bulk self-trapped excitons of TiO_2_.^38^ A decrease in PL emission is observed when nickel species are added to TiO_2_, suggesting that electron/hole recombination is suppressed. This feature is crucial for photocatalytic reactions since reactive radicals are prone to interact with substrates.

### Methane Photooxidation Mechanism

As described earlier, Ni–TiO_2_ was the best photocatalyst for methane oxidation to produce methanol. The introduction of nickel changes the electronic structure of TiO_2_, as shown by the PL results. Due to the excellent performance of the material containing 0.5% Ni, a more in-depth analysis was conducted to understand the electronic properties of this material. Figure 3a clearly illustrates the varied behaviors exhibited by different metal–TiO_2_ materials in methane photooxidation. Noble metals likely generate •OOH upon interaction with peroxide molecules, leading to the production of CH_3_OOH, which can subsequently decompose into methanol. However, Ag and Pd–TiO_2_ exhibit nonselective behavior, generating CO_2_ as a byproduct. In contrast, non-noble metals such as Cu and Co–TiO_2_ demonstrate markedly different activities compared to Ni–TiO_2_. This distinction can be attributed to the unique charge transfer dynamics inherent in these materials. Previous studies have reported that CoO_*x*_ on TiO_2_ tends to receive photogenerated holes rather than electrons,^20^ a phenomenon similarly observed in Cu species on Cu–W–TiO_2_ photocatalysts.^19^ These findings suggest that Ni–TiO_2_ displays distinct behavior, rendering it more active in the methane photooxidation process.

**Figure 8:**
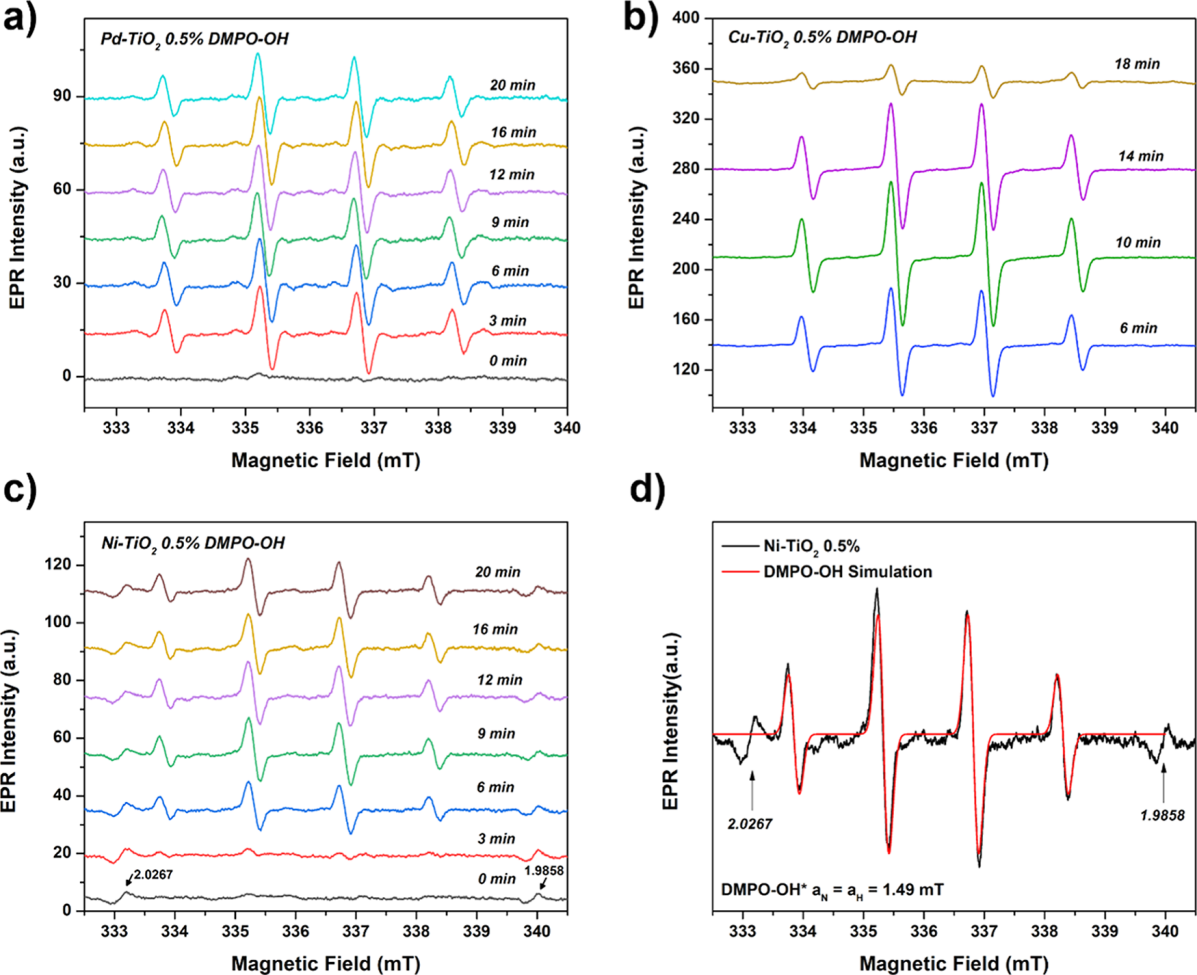
EPR spin trapping analysis using DMPO in H_2_O to probe the formation of •OH radicals for (a) Pd–TiO_2_, (b) Ni–TiO_2_, and (c) Cu–TiO_2_. (d) Simulation of DMPO-OH signals and experimental spectra obtained with the Ni–TiO_2_ photocatalyst.

To understand the mechanism of all catalysts, we performed EPR assays to detect radicals in the reaction system. The five photocatalysts were divided into three different groups: noble metals (Ag and Pd), non-noble metals (Cu and Co), and Ni–TiO_2_. First, we performed solid-state EPR and verified that the metal catalysts exhibit similar defects (Figure S11a), characterized by the presence of Ti^3+^ and single-electron-trapped oxygen vacancies (SETOVs) (Figure S11b).^39^ These defects are promoted by metal doping, which can enhance charge separation and adsorption of oxygen species.^40^ EPR analysis was also used to identify radicals produced on reaction solution. According to Figure 8a–c, the three materials basically produced the same reactive species in H_2_O. The spin adducts, characteristic of the capture of the reactive oxygen species by the spin trap, were identified by the quartet signal of intensity 1:2:2:1, with the hyperfine parameters *a*_N_ = *a*_H_ = 1.49 mT, and a triplet of the same intensity with the separation between the transitions of the order of 1.46 mT. The first signal is characteristic of the spin adduct generated by the reaction between DMPO and hydroxyl radicals (•OH).^41^ In contrast, the three transition lines are generated due to the spontaneous degradation of DMPO when subjected to light (Figure S12).^42^ The comparison of Pd, Ni, and Cu metals for hydroxyl radical production reveals distinct behaviors. When observing the evolution of DMPO-OH signals generated by Pd–TiO_2_ (Figure 8a), it becomes evident that a higher quantity of radicals is produced even at shorter times (3 min). This characteristic indicates that Pd atoms induce elevated levels of •OH radicals, highlighting how noble metals enhance the reduction of H_2_O_2_ molecules. Moreover, such high levels of hydroxyl radicals may account for the subsequent overoxidation to CO_2_ observed in these photocatalysts. Cu–TiO_2_ (Figure 8b) exhibits a decrease of •OH radical production at longer times (20 min), suggesting a limited capability to reduce peroxide molecules. Ni–TiO_2_, conversely, possesses a controlled •OH radical, which intensifies over the course of the reaction (Figure 8c). Furthermore, it is possible to observe signals for Ni–TiO_2_ with g values of 1.98 and 2.02 (Figure 8c,d), which can be related to Ti^3+^ at lattice sites and superoxide radical anions (O_2_^•–^).^22,43^

**Figure 9:**
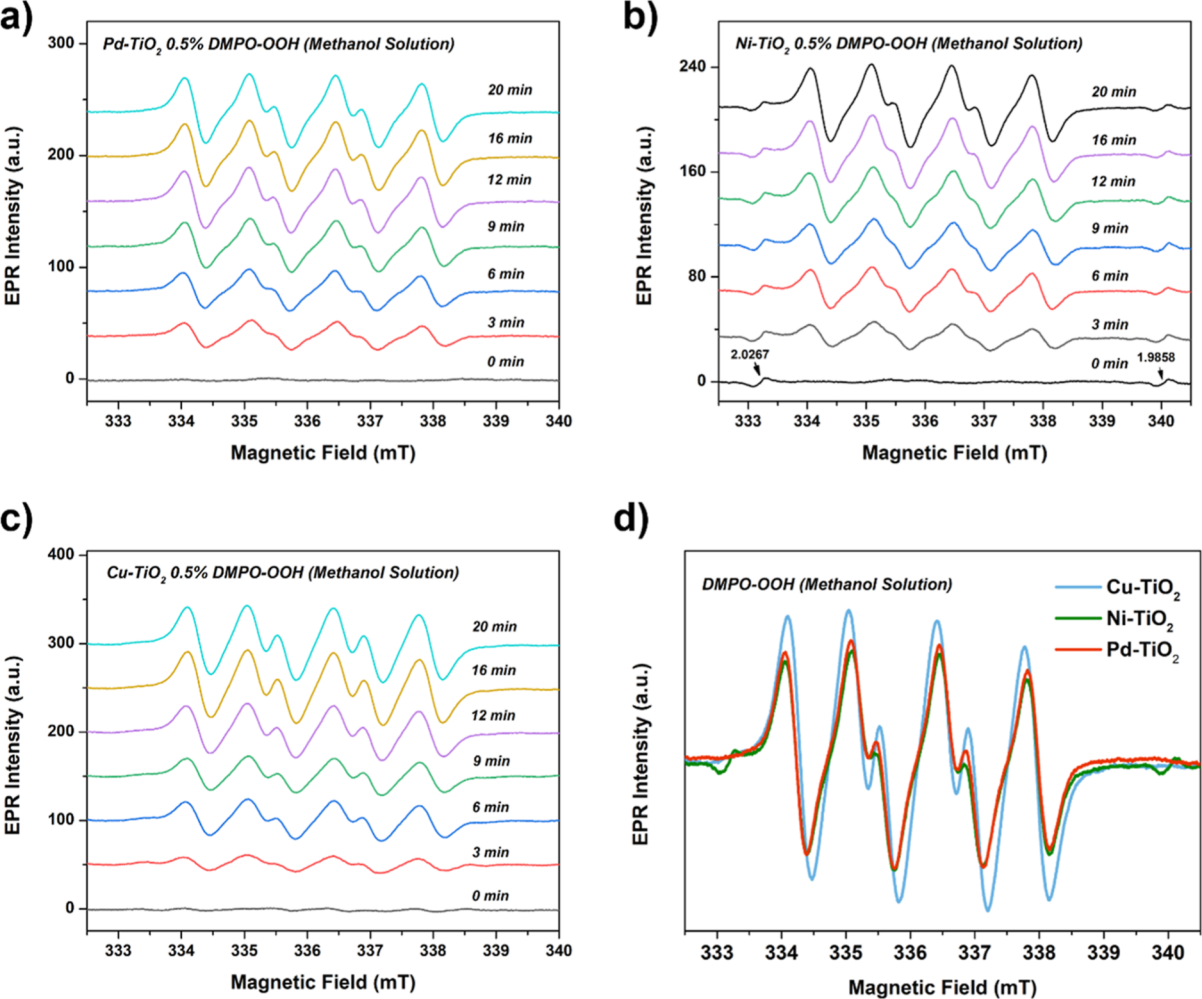
EPR spin trapping analysis using DMPO in methanol to probe the formation of •OOH radicals for (a) Pd–TiO_2_, (b) Ni–TiO_2_, and (c) Cu–TiO_2_. (d) Comparison of DMPO-OOH signals for Cu, Ni, and Pd–TiO_2_ materials at 20 min of irradiation.

Furthermore, the presence of superoxide radicals (O_2_^•–^) can be verified using a methanol solution.^44^ These radicals typically appear in the form of •OOH in protic media. Figure 9 demonstrates that all photocatalysts produce •OOH radicals (simulated spectra in Figure S13). The production of superoxide species can occur through several pathways, but since there is no O_2_, the most likely pathway involves the oxidation of H_2_O_2_ by photogenerated holes. While the production of •OH radicals remains constant, we observed a gradual increase in the intensity of DMPO-OOH signals for Pd–TiO_2_ (Figure 9a), similar to the trend observed for Ni–TiO_2_ (Figure 9b). This trend suggests that the formation of superoxide species may occur at a similar level for both materials. For Cu–TiO_2_, it is evident that this material promotes the formation of •OOH species (Figure 9c,d), which are generated by the oxidation of H_2_O_2_. This outcome suggests that copper species enhance oxidation reactions, indicating that photogenerated holes are transferred to Cu sites, consistent with previous studies.^19^ Moreover, to enhance our discussion, we conducted a fluorescence analysis using terephthalic acid (TPA) as a probe molecule for hydroxyl radicals. As shown in Figure S14, in the absence of H_2_O_2_, only the Ni–TiO_2_ photocatalyst is capable of producing OH radicals through H_2_O-oxidation. This outcome can be attributed to the fact that the valence band of CuO cannot oxidize water.^45^ This finding suggests that photogenerated holes in TiO_2_ are transferred to the valence band of the CuO_*x*_ sites. A similar trend can be extended to Co–TiO_2_, which exhibits the same characteristic as reported in other works.^46^ The limited activity for oxygenate production by the photocatalysts can be attributed to the competition between CH_4_ and H_2_O_2_ for oxidation sites, as well as the low production of •OH due to the poor efficiency of Ti^3+^ sites in reducing peroxide molecules.

**Figure 10:**
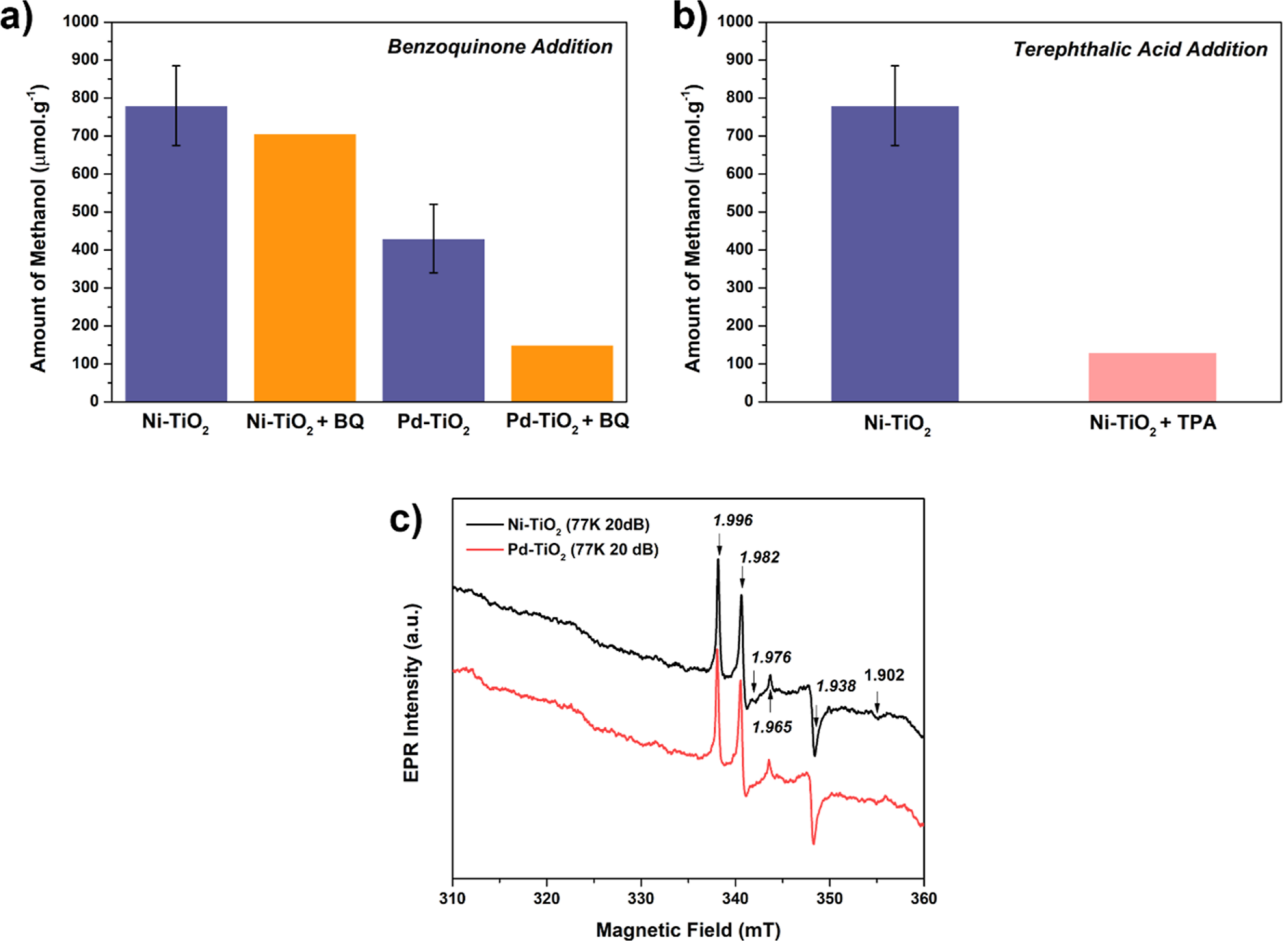
Photocatalytic methane oxidation reactions using (a) *p*-BQ as a superoxide scavenger and (b) TPA as a hydroxyl radical scavenger (2 mM H_2_O_2_, 2 h). (c) Solid-state EPR spectra of Ni and Pd–TiO_2_ recorded at 77 K (at these conditions, it is not possible to observe SETOVs because they are saturated).

The reasons behind the low activity of the Cu and Co–TiO_2_ photocatalysts for CH_4_ oxidation are apparent. However, understanding the disparity in reactivity between noble metals (Ag and Pd) and NiO_*x*_ clusters remains unclear, despite both materials producing the same radicals. One possible explanation lies in the abundant hydroxyl radicals produced by Pd–TiO_2_. Another aspect to consider is the role of •OOH radicals in this reaction. To study the influence of different radicals in the production of oxygenates, we conducted a photocatalytic reaction using benzoquinone (BQ) and TPA as scavengers for superoxide and hydroxyl radicals, respectively.^19^ As shown in Figure 10a, the addition of BQ to Ni–TiO_2_ resulted in a decrease in methanol production within the error margins, suggesting that •OOH radicals are not essential for the mechanism. However, when BQ was introduced in the reaction with Pd–TiO_2_, methanol production decreased significantly, and CH_3_OOH was not produced as a byproduct. This finding strongly suggests that superoxide radicals are crucial for methanol production with Pd sites but not with Ni clusters. Furthermore, the addition of TPA to the reaction with Ni–TiO_2_ led to a substantial decrease in the methanol yield (Figure 10b), confirming that •OH radicals are primarily responsible for CH_3_OH production.

It may be possible that noble metals can stabilize •OOH radicals for longer periods, thereby allowing the production of CH_3_OOH. This notion is supported by other studies using Pd as cocatalysts, which have reported the production of CH_3_OOH molecules.^26,47^ In contrast, superoxide radicals do not participate in the mechanism of Ni–TiO_2_. Superoxide radicals are known to be more unstable than hydroxyl radicals,^48^ suggesting that they react quickly before reaching methyl radicals. Low-temperature EPR measurements indicate the presence of several signals related to Ti^3+^ defects on Ni and Pd–TiO_2_ (Figure 10c). These signals (*g*-factor shown in the inset of the graph) are characteristic of the paramagnetic center Ti^3+^ in different chemical environments: 1.98 and 1.92 represent this species on the semiconductor surface, while 1.99 and 1.96 correspond to the *g*_⊥_ and *g*_||_ components of the Ti^3+^ in the bulk.^49^ This Ti 3d^1^ state can act as a trap site for electrons, which generally lies below the conduction band.^50^ These electrons can be easily transferred to Pd or Ni sites, causing a synergistic improvement of reductive reactions.

**Figure 11:**
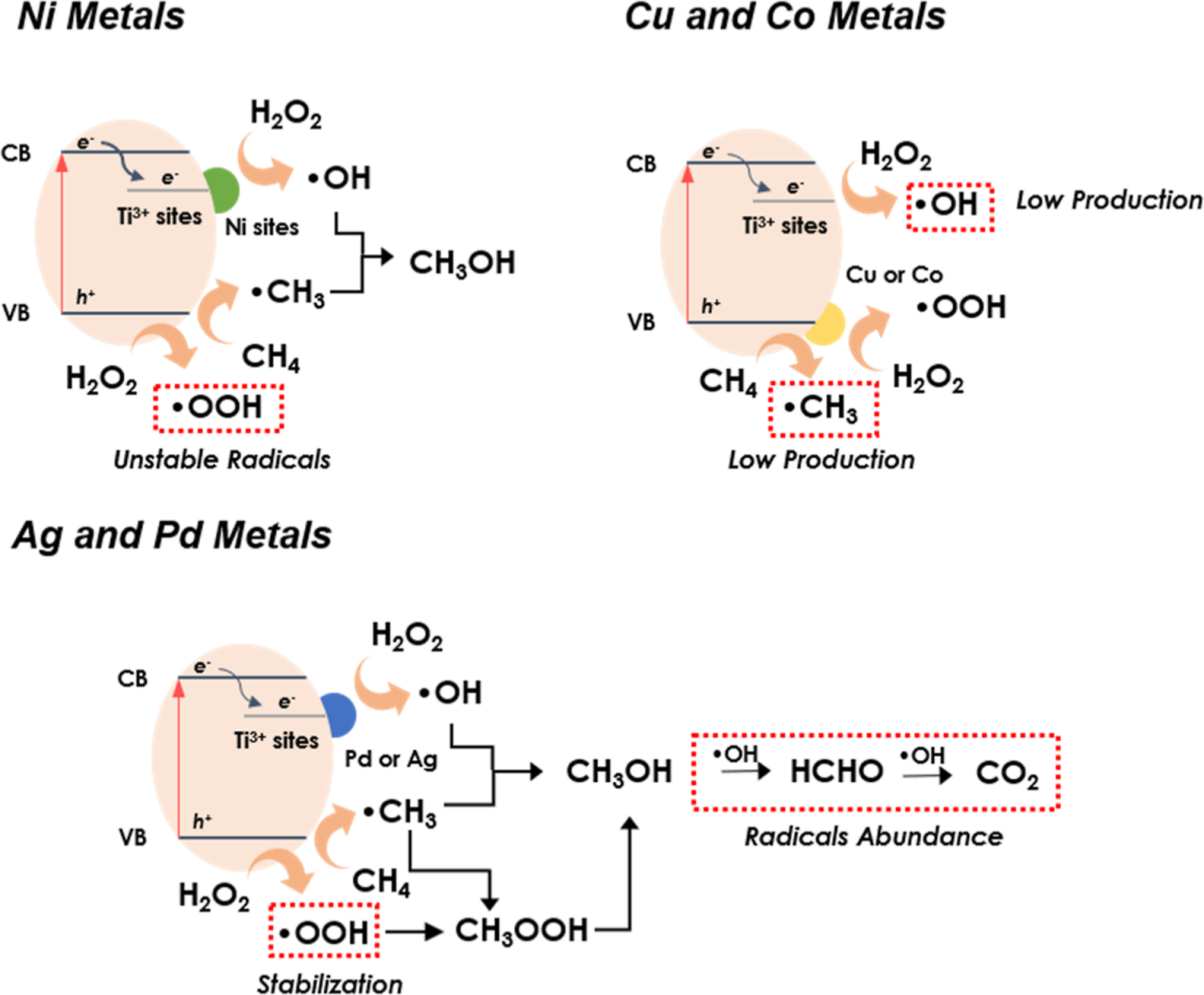
Schematic representation of the metal–TiO_2_ mechanisms for photocatalytic CH_4_ oxidation.

The proposed reaction mechanisms for the various metal–TiO_2_ catalysts are illustrated in Figure 11, where the three categories of photocatalysts are delineated. In contrast to Cu and CoO_*x*_ sites, our findings suggest that NiO_*x*_ clusters facilitate reduction reactions, indicating that these sites receive photogenerated electrons. On the other hand, Ag and Pd metals follow a distinct pathway, with •OOH radicals playing a pivotal role in the production of methanol. Regarding Ni and noble metals, it is expected that CH_4_ molecules are activated on oxygen vacancies (SETOVs), consistent with findings from previous studies.^22,51^

**Figure 12:**
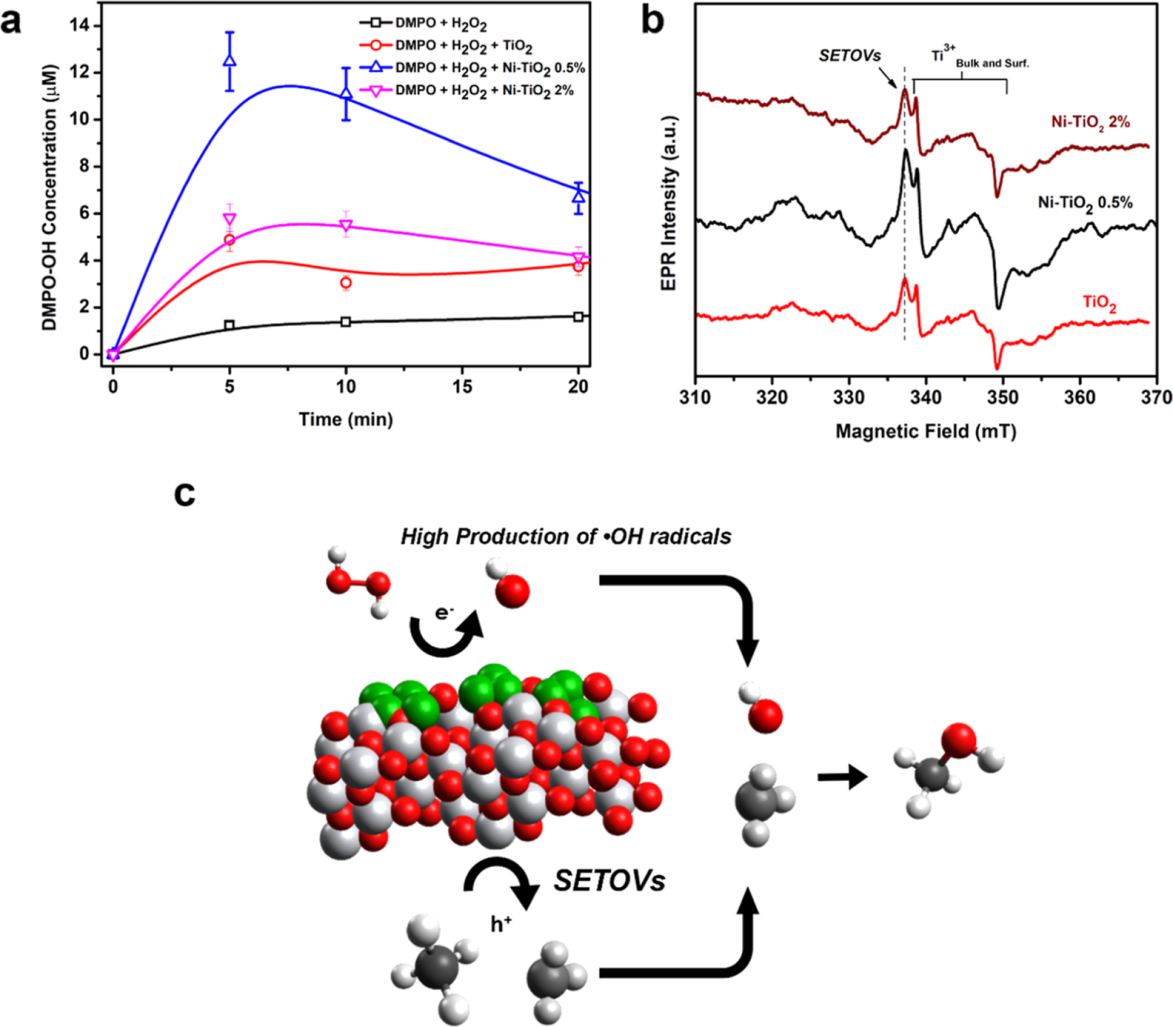
(a) Production kinetics of the EPR spin adducts generated between the reaction of the hydroxyl radical and the DMPO spin trap; (b) powder EPR spectra of Ni–TiO_2_ and TiO_2_ catalysts; and (c) photocatalytic mechanism for methane oxidation with Ni–TiO_2_.

The influence of nickel loading on the photocatalytic activity was further evaluated. Upon comparing the kinetics of spin adduct generation (DMPO/•OH) among the Ni–TiO_2_ samples (Figure 12a), we can affirm that the Ni–TiO_2_ 0.5% sample produced the highest quantity of hydroxyl radicals, while pure TiO_2_ exhibited the worst performance. Furthermore, all materials generated more spin adducts than in the absence of the photocatalyst (DMPO only), indicating that the presence of the catalyst improves H_2_O_2_ utilization. After 10 min of UVA radiation, the adducts began to degrade due to the oxidative nature of the medium and the instability of these paramagnetic species. Summarizing the results from Figure 12b, it is evident that Ni sites exhibit high selectivity in producing hydroxyl radicals to produce CH_3_OH molecules. As mentioned before, the activity of metal–TiO_2_ is directly related to the presence of defects in the structure. When we compare solid-state EPR spectra of Ni–TiO_2_ at different loadings, it is clear that Ni–TiO_2_ 0.5% possesses the higher presence of defects, including Ti^3+^ sites and SETOVs. Specially, the presence of these oxygen vacancies in the structure is essential for CH_4_ activation to produce methyl radicals. Since Ni–TiO_2_ 0.5% has the highest presence of SETOVs, this result can be correlated to the high production of methanol by this photocatalyst. Overall, Ni–TiO_2_ presents an excellent performance for CH_4_ oxidation due to its unique mechanism between other metals and high production of hydroxyl radicals (Figure 12c).

## Conclusions

In summary, our investigation highlights Ni–TiO_2_ as the most effective photocatalyst for methane photooxidation to methanol among various metal-supported TiO_2_ materials, when hydrogen peroxide (H_2_O_2_) is used as the oxidant, with minimal CO_2_ evolution. Ni–TiO_2_ 0.5% exhibited remarkable methanol production, reaching 1,600 μmol·g^–1^ after 4 h and achieving a 10% methane conversion under UV irradiation. The results indicate that distinct mechanisms are observed among the metal–TiO_2_ photocatalysts. Unlike noble metals, Ni sites promote a pathway in which superoxide radicals are not involved, thereby enhancing the selectivity and reducing overoxidation to CO_2_. Moreover, the high presence of structural defects, such as Ti^3+^ and SETOVs, in Ni–TiO_2_ of 0.5% ensures its superior CH_3_OH production. This study elucidates how distinct metal sites can act in varying ways, influencing the production of different reaction products.

## Experimental Section

### Photocatalysts Synthesis

Photocatalysts based on TiO_2_ with different proportions of metals (0.5, 1, and 2%) were prepared using the impregnation method. For this, a certain amount of metal chloride (e.g., FeCl_3_, NiCl_2_, among others) was dissolved in 2 mL of deionized water and then added dropwise in 1 g of commercial TiO_2_ anatase (TiO_2_, Aldrich, 99.8%), previously dissolved in 10 mL of deionized water, under vigorous stirring on a heating plate at 100 °C. After the total evaporation of water, the material was transferred to an alumina crucible and placed in a muffle furnace (ROTINA 380R, Hettich). Then, it was heated at a rate of 5 °C/min and kept at 400 °C for 4 h. After cooling, the material was stored for characterization and photocatalytic activity testing.

### Characterization

The crystalline phases of the photocatalysts were characterized by powder XRD using a Shimadzu XRD-6000 diffractometer, operating with CuKα radiation (λ = 0.15406 nm), with a rate of 0.5° min^–1^ in the range of 2θ = 10 to 80°. The crystallite sizes for TiO_2_ were estimated by using the Scherrer equation (eq 1).
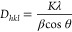
1where *D*_*hkl*_ is the crystallite size (nm), β corresponds to the full width at half-maximum (fwhm) of the diffraction peak, *K* is the constant (0,9), λ is the wavelength of the X-ray source (CuKα 1.5406 Å), and θ is Bragg’s angle.

Raman spectroscopy was performed at room temperature using a LabRAM microspectrometer (Horiba Jobin-Yvon) equipped with an Olympus TM BX41 microscope with He–Ne laser (λ = 512 nm and 5.9 mW).

The amounts of Ni, Co, Cu, and Ag in the samples were determined by flame atomic absorption spectroscopy using a PerkinElmer PinAAcle 900T model. The flame consisted of synthetic air (10 mL) and acetylene (2.5 mL) at a wavelength of 324.75 nm. Band gaps were determined by the Tauc method from diffuse reflectance spectra in a Shimadzu UV-2600 equipment, in the ultraviolet–visible region. The BET method was used to calculate the specific surface area values. For this, a micromeritics ASAP 2020 analyzer at 77 K was used. Samples were previously degassed at 80 °C under vacuum until a degassing pressure < 10 μmHg was reached.

To verify the oxidation state of Ni species on TiO_2_ and the surface composition of the Ni–TiO_2_ sample, XPS was performed by using a ScientaOmicron ESCA + spectrometer and a high-performance hemisphere analyzer (EAC2000 sphere) equipped with a monochromator and Al Kα source (*h*ν = 1486.6 eV). All spectra obtained were calibrated using the binding energy of the adventitious carbon bond (C–C), set at 284.8 eV.

TEM images were acquired using the Thermo Fisher/FEI Titan Cubed Themis microscope, double-corrected and equipped with a monochromator, operated at 300 kV and equipped with a 4kx4k CMOS type Ceta camera. Analyses were performed using conventional transmission electron microscopy (CTEM) with a parallel and convergent beam in STEM mode, and images were collected using high angle annular dark field and bright field detectors. Chemical composition mapping was carried out using energy-dispersive X-ray (EDX) spectroscopy operated in STEM mode. The grids were prepared by depositing a small aliquot of 3 μL of a previously sonicated sample suspension onto a 400-mesh copper grid coated with an ultrathin carbon film. The grid was allowed to dry at room temperature.

### Photocatalytic Tests

Methane photo-oxidation tests were carried out in a quartz tube (140 mL) illuminated with 6 UV lamps (Osram, 15 W, 254 nm) (Figure S15). The reaction temperature was maintained at 25 °C using a thermostatic bath (SolidSteel). In each test, 100 mg of photocatalyst was added to a hydrogen peroxide solution (NEON, 35% P.A.) in deionized water. In order to saturate the reactor, CH_4_ (99.9%) was bubbled into the suspension with a constant flow for 15 min. The production of CO_2_ and CO was analyzed at the end of the reaction in a gas chromatograph (Thermo CP-3800) equipped with a flame ionization detector and a thermal conductivity detector with a packed HayeSep N column (0.5 m × 1.8 in.) and a 13X molecular sieve column (1.5 m × 1.8″). Argon was used as the carrier gas, and the methanizer temperature was 350 °C. The calibration curve of CO_2_ measured by GC-TCD is displayed at Figure S16.

Liquid products were quantified by ^1^H NMR (600 MHz, Ascend 600 Bruker) at 25 °C. For each test, 540 μL of the sample was mixed with 60 μL of D_2_O solution containing 5.0 mM dimethyl sulfoxide (DMSO) as a standard and 0.21 mM TSPd_4_ as a reference. A WET procedure suppressed the water peak. NMR data were processed using MestReNova software. Representative ^1^H NMR spectra used to determine and calculate the concentration of liquid products are shown in the main text.

The quantification of liquid products by ^1^H NMR for compounds was calculated from eq 2.
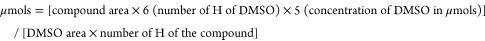
2

To confirm the quantification of NMR experiments, methanol was also quantified by GC-FID using a DB-WAX column and He as the carrier gas. For each test, 150 μL of reaction sample was mixed with 50 μL of a 1-octanol solution (1.5 mM) in high-purity CH_3_CN (99.99%) (Figure S17). To ensure that methanol was present only in the reaction sample, blanks with deionized water and the external standard were injected. The calibration curve of methanol using GC-FID is shown at Figure S18. A comparison between some results obtained from NMR and GC methods is organized in Table S4.

The concentration of liquid formaldehyde (HCHO) was quantified using a colorimetric method described elsewhere.^52^ An aqueous solution (100 mL) was prepared by dissolving 15 g of ammonium acetate, 0.3 mL of acetic acid, and 0.2 mL of pentane-2,4-dione. Then, 0.5 mL of the reaction liquid product was mixed with 2.0 mL of water and 0.5 mL of reagent solution. The mixed solution was kept at 35 °C and measured by UV–Vis at 412 nm. The concentration of HCHO in the liquid product was determined by the calibration curve (Figure S19).

Methane conversion was calculated based on the sum of methane in liquid (dissolved in water) and gaseous phases. Liquid methane was extracted from Duan and Mao’s study,^200^ which predicts that the solubility of methane in pure H_2_O is 0.00126 mol·kg^–1^. Since 100 mL of water was used at methane oxidation reactions, we can assume that 126 μmol of CH_4_ is present in the liquid phase. Methane in the gaseous phase was calculated based on STP conditions, i.e., 1 mol of gas at 25 L. Therefore, 1600 μmol of CH_4_ was considered in 40 mL of headspace. Methane conversion was calculated from eq 3
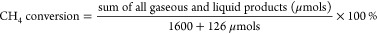
3

### PL Probe Assays

The production of hydroxyl radicals of Ni–TiO_2_ and Cu–TiO_2_ using only H_2_O and UV radiation was measured by the PL probe method with TPA. When •OH radicals are formed in solution with TPA, 2-hydroxyterephthalic acid (HTPA), a fluorescent compound, is produced. In each test, 50 mg of photocatalyst was placed with a mixture of 20 mL of TPA solution (0.5 mM) and 80 mL of NaOH (2 mM) solution. The tests were performed with the same reaction system mentioned above without H_2_O_2_ addition. The HTPA concentration was monitored by fluorescence measurements using a spectrofluorophotometer (Shimadzu RF-5301PC). The fluorescence emission spectrum was obtained by using excitation at 315 nm.

### EPR Measurements

EPR measurements allied to spin trapping methodology were conducted using 5,5-dimethyl-1-pyrroline *n*-oxide (DMPO, CAS 3317-61-1, 96%, Oakwood, EUA) and *N*-*tert*-butyl-α-phenylnitrone (PBN, CAS 3376-24-7, 98%, TCI America, Japão). For the measurements, a Magnettech Mini Scope MS400 EPR X-Band spectrometer was used operating with the following configurations: 10 mW microwave power, 100 kHz modulation field with 0.2 mT amplitude, 337 mT centered field, 60 s scan time, and 4096 integration points. Low-temperature measurements were performed on another MiniScope 400 EPR spectrometer modified by an ESR 900 cryosystem (Oxford Instruments, United Kingdom) using a liquid helium flow. The temperature was controlled by a MercuryIC (Oxford Instruments, United Kingdom).

For the spin trapping experiments with DMPO, 20 mg of this spin trap was solubilized in 1 mL of solvent: (i) deionized water to detect •OH radicals or (ii) methanol solution (1.5 mL) to observe O_2_^•–^ radicals. In these solutions, 5 mg of photocatalyst was suspended and 6 μL of H_2_O_2_ (30% V/V) was added, and the system was illuminated with a UVA lamp with an irradiance of 16 mW·cm^–2^. Aliquots were removed with the aid of a glass capillary (∼50 μL) and placed in a quartz tube (Wilmad-Labglass, United States), which was then inserted into the cavity of the EPR spectrometer. The adducts were simulated by using EasySpin.

## Supporting Information Available

Fabrication of photocatalysts; characterization; photocatalytic tests; EPR measurements; XRD powder patterns of metal–TiO_2_ samples; evolution of the CO_2_ concentration along the reaction time for Ni–TiO_2_ 0.5%; diffuse reflectance UV–vis of the materials; SEM of samples TiO_2_, Ni–TiO_2_ 0.5%, and Ni–TiO_2_ 2%; TEM pictures and histogram of particle size distribution of TiO_2_ and Ni–TiO_2_ 0.5%; SAED of the Ni–TiO_2_ and TiO_2_; survey spectra of Ni–TiO_2_ 0.5% (a) and Ni–TiO_2_ 2%; Raman spectra of Ni–TiO_2_ and TiO_2_ (a) and Raman shift; experimental setup for the methane photooxidation reactions; GC-FID chromatogram of methanol and ethanol; calibration curve of methanol calculated by GC-FID; calibration curve of CO_2_ calculated by GC-TCD; chromatogram of GC-TCD showing peaks for Ni–TiO_2_ methane photooxidation reaction; and FHWM and mean crystallite size (D-Scherrer) for TiO_2_ calculated by the Scherrer equation (PDF)
