# Supplementary material for: Investigating the Metal–TiO2 Influence for Highly Selective Photocatalytic Oxidation of Methane to Methanol
Source: ACS Appl Mater Interfaces. 2024 Jul 23;16(32):41973–85. doi: 10.1021/acsami.4c02862 (PMC11331436; doi:10.1021/acsami.4c02862)
Supplement: Supplementary file 1 — am4c02862_si_001.pdf [file am4c02862_si_001.pdf]

Supporting Information

Investigating the Metal-TiO_2 Influence for Highly Selective Photocatalytic Oxidation of Methane to Methanol

*Marcos A. R. da Silva,[1,2] Jéssica C. Gil,[1,2] Juliana A. Torres,[1] Gelson T. S. T. Silva,[1] José Balena Gabriel Filho,[3] Henrique Fernandes Vieira Victória,[3] Prof. Klaus Krambrock,[4] Ivo F. Teixeira[1] and Caue Ribeiro[1,2]**

[1] Nanotechnology National Laboratory for Agriculture (LNNA), Embrapa Instrumentation, São Carlos 13561-206, Brazil

[2] Department of Chemistry, Federal University of São Carlos (UFSCar), 13565-905, São Carlos - São Paulo, Brazil

[3] Department of Chemistry, Federal University of Minas Gerais (UFMG), 31270-901, Belo Horizonte, MG, Brazil

[4] Department of Physics, Federal University of Minas Gerais (UFMG), 31270-901, Belo Horizonte, MG, Brazil

* Corresponding author: caue.ribeiro@embrapa.br

Tel.: +55 16 2107 2800; fax: +55 16 2107 2902

SUPPLEMENTARY INFORMATION

FIGURES

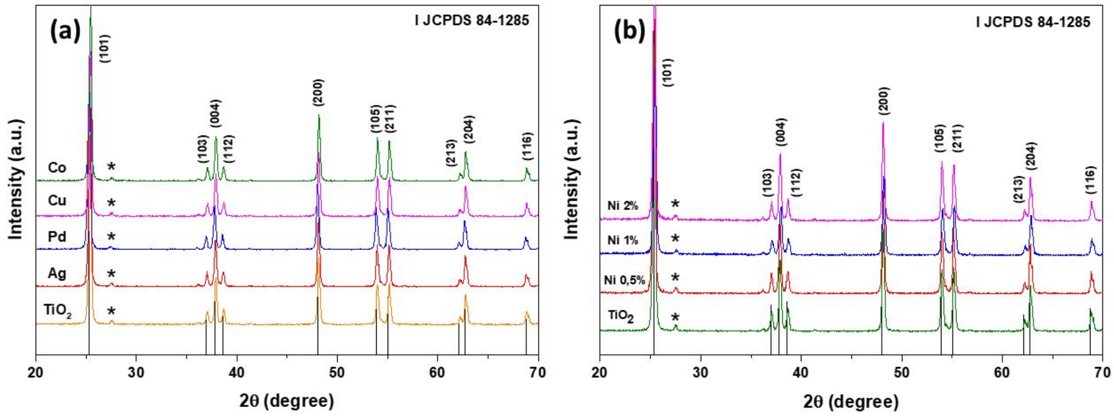

Figure S1. XRD powder patterns of as-prepared samples. **(a)** TiO_2 loaded with different metals with concentrations of 0.5% and **(b)** TiO_2 loaded with different concentrations of Ni. *Diffraction peak related to the rutile TiO_2 phase (JCPDS 84-1285).

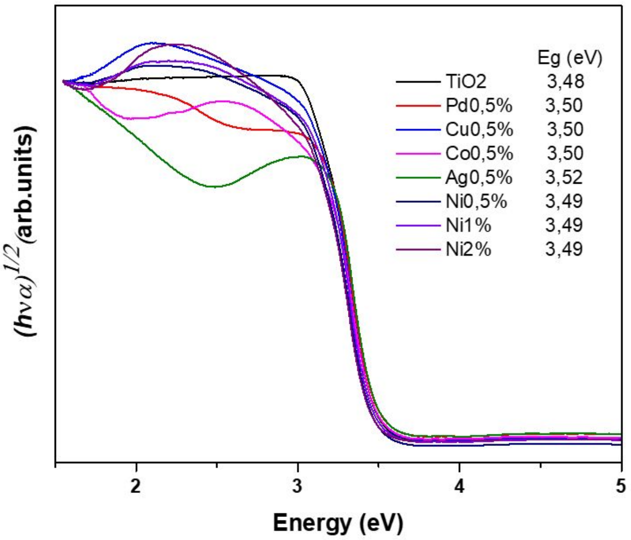

Figure S2. UV-Vis diffuse reflectance of metal-TiO_2 materials.

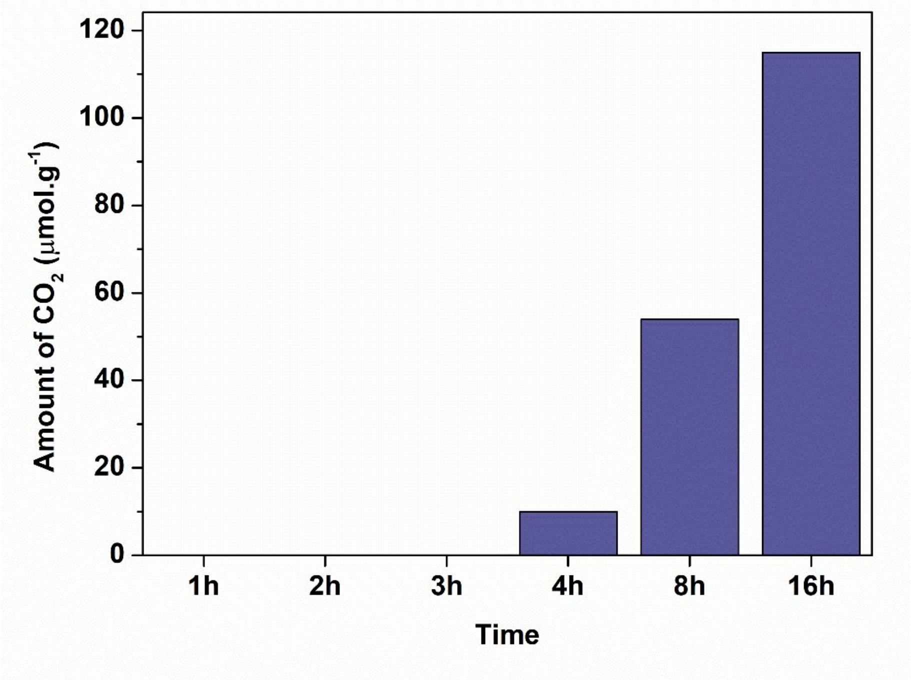

Figure S3. Evolution of the CO_2 concentration along the reaction time for Ni-TiO_2 0.5%. Reaction conditions: 100 mg of photocatalysts, 2 mM H_2O_2 in 100 mL of H_2O and CH_4 (99.9%) operated at room temperature and atmospheric pressure.

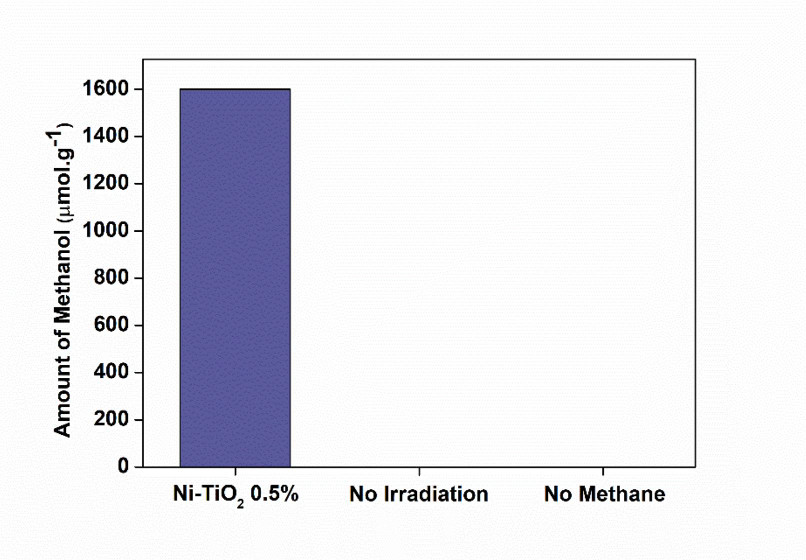

Figure S4. Comparison of methanol yield of Ni-TiO_2 with reaction carried out without irradiation or methane. Reaction conditions: 100 mg of photocatalyst, 2 mM H_2O_2 in 100 mL of H_2O and CH_4 (99.9%) operated at room temperature and atmospheric pressure.

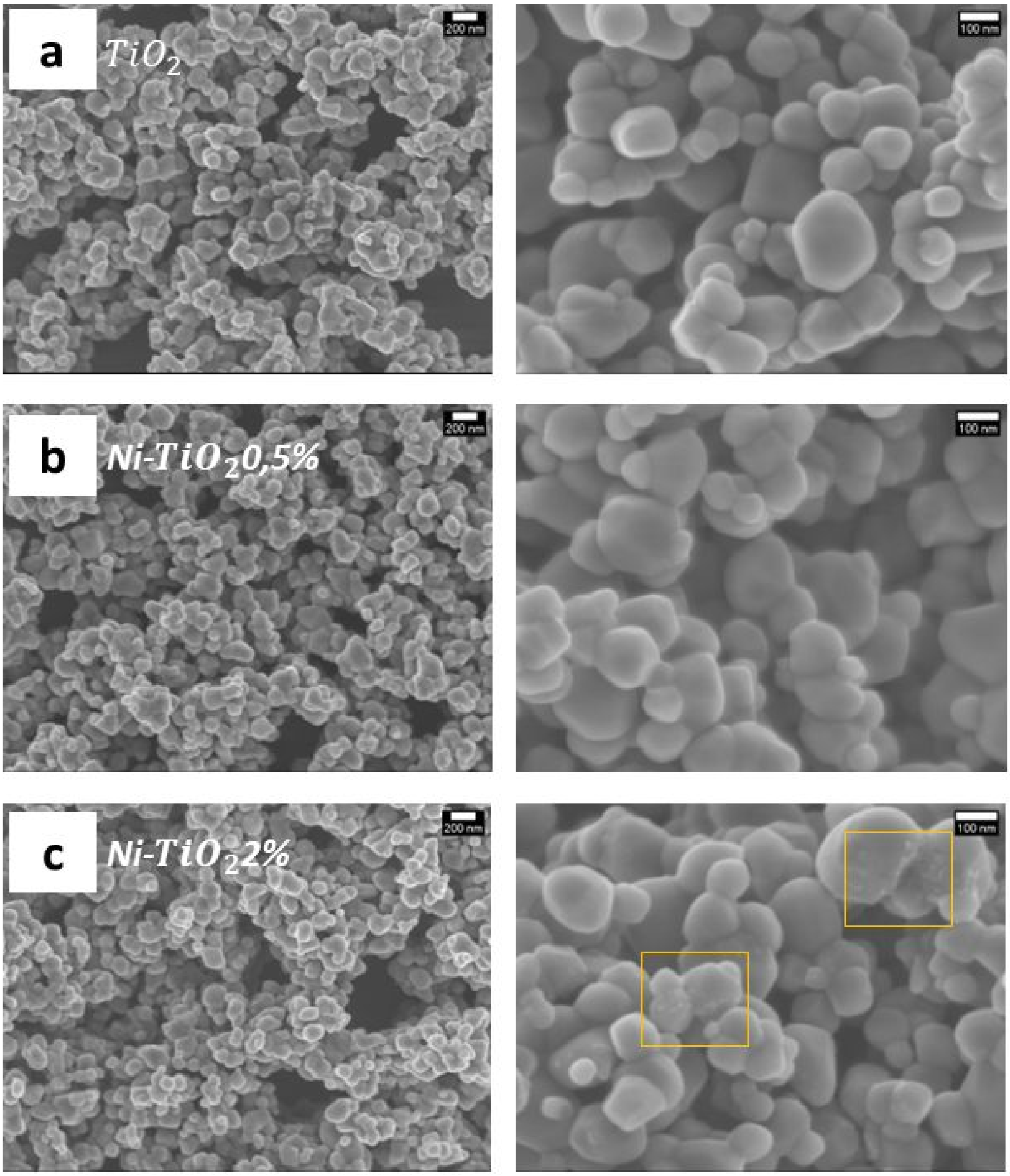

Figure S5. SEM of samples **(a)** TiO_2, **(b)** Ni-TiO_2 0.5% and **(c)** Ni-TiO_2 2%. It is possible to observe individual particles with expected dimensions (40-300 nm). The image **(c)** shows in the highlight **(in yellow)** surface roughness, possibly originated from the greater amount of nickel present in the sample.

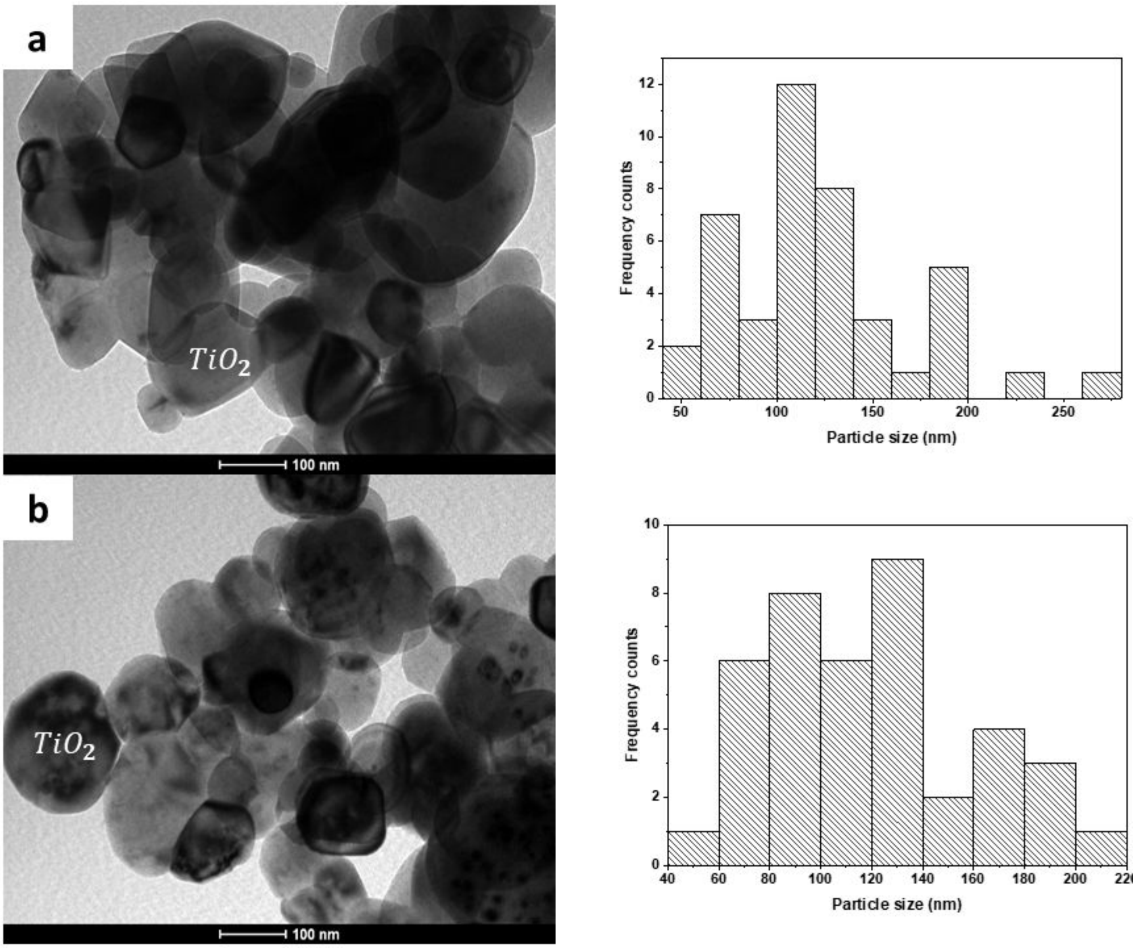

Figure S6. TEM images and histogram of particle size distribution of **(a)** TiO_2 and **(b)** Ni-TiO_2 0.5%.

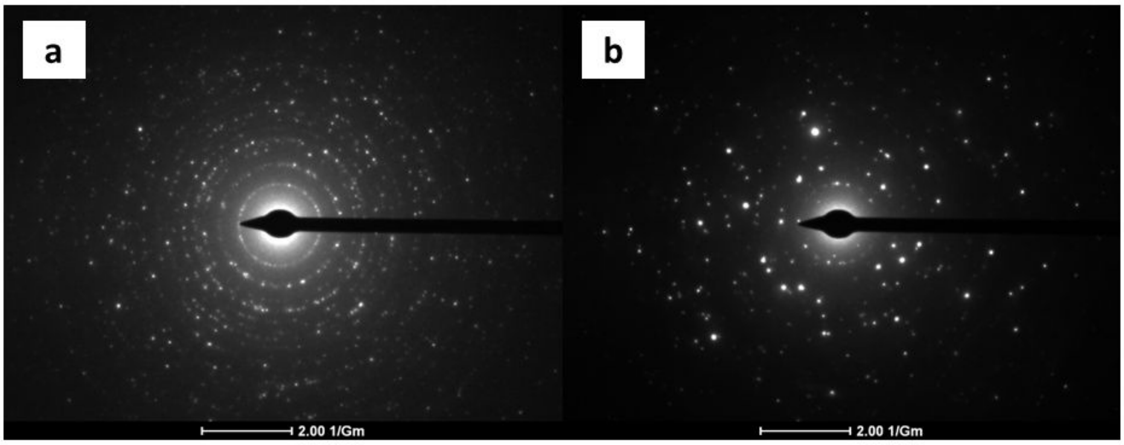

Figure S7. Selected-area electron diffraction (SAED) patterns of **(a)** TiO_2 and **(b)** Ni-TiO_2 0.5%.

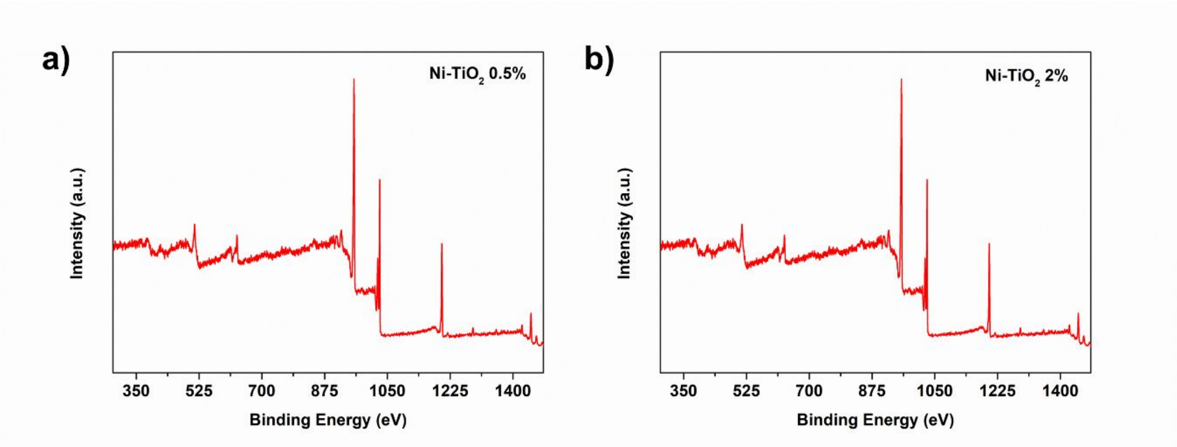

Figure S8. Survey spectra of **(a)** Ni-TiO_2 0.5% and **(b)** Ni-TiO_2 2%.

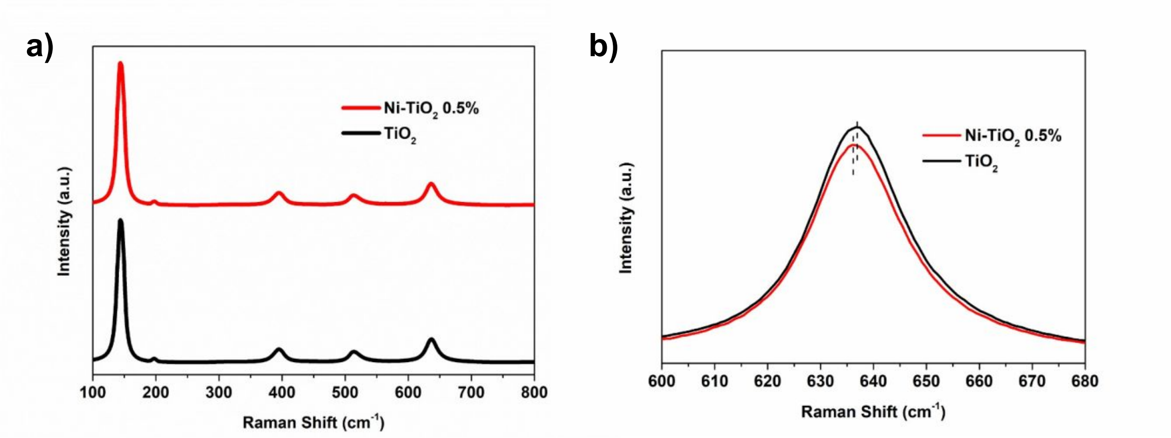

Figure S9. Raman spectra of **(a)** Ni-TiO_2 0.5% and TiO_2 **(b)** and Raman slight shift.

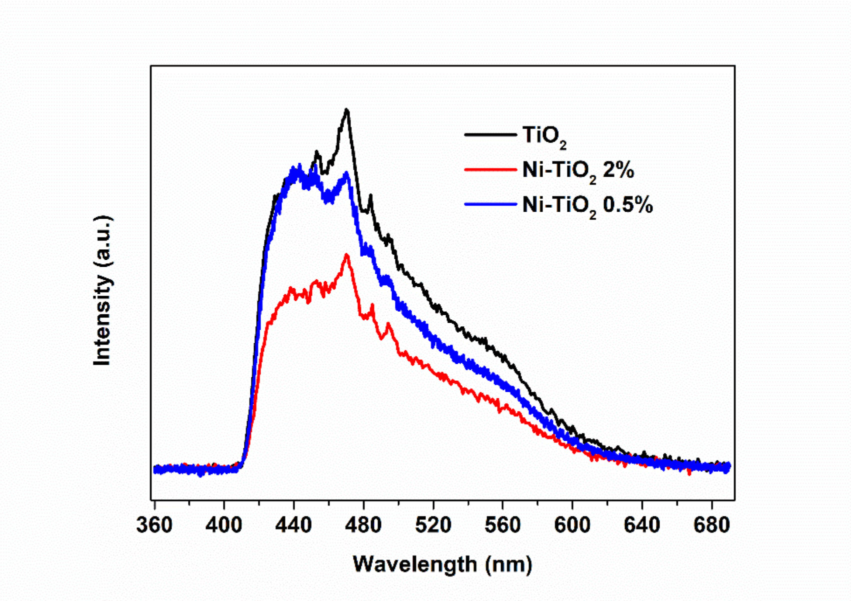

Figure S10. Photoluminescence emission spectra of the TiO_2, Ni-TiO_2 0.5% and Ni-TiO_2 2%.

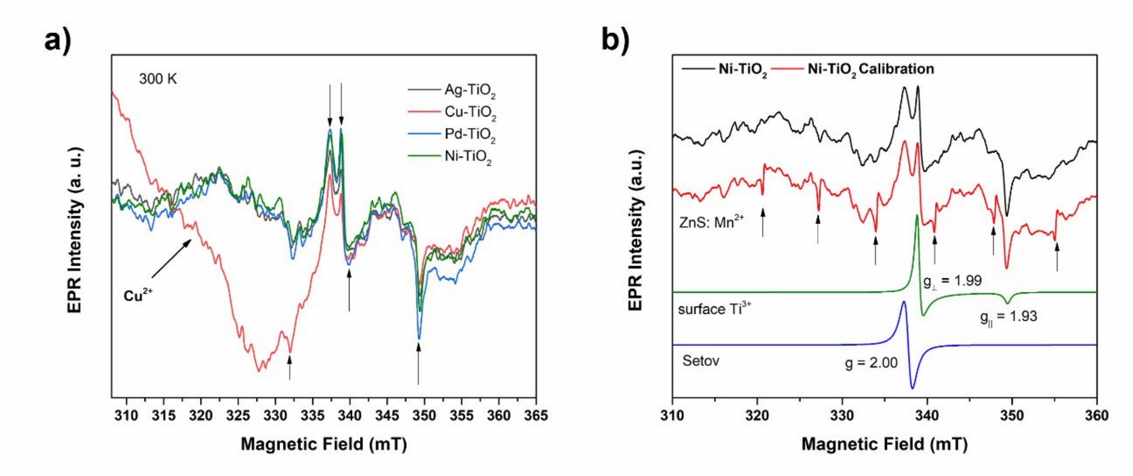

Figure S11. (a) Solid state EPR spectra of different metal-TiO_2 materials and **(b)** experimental and simulated spectra of solid-state measurements at room temperature. The red spectrum was recorded using a Mn^{2+} (in ZnS) standard for g-factor calibration.

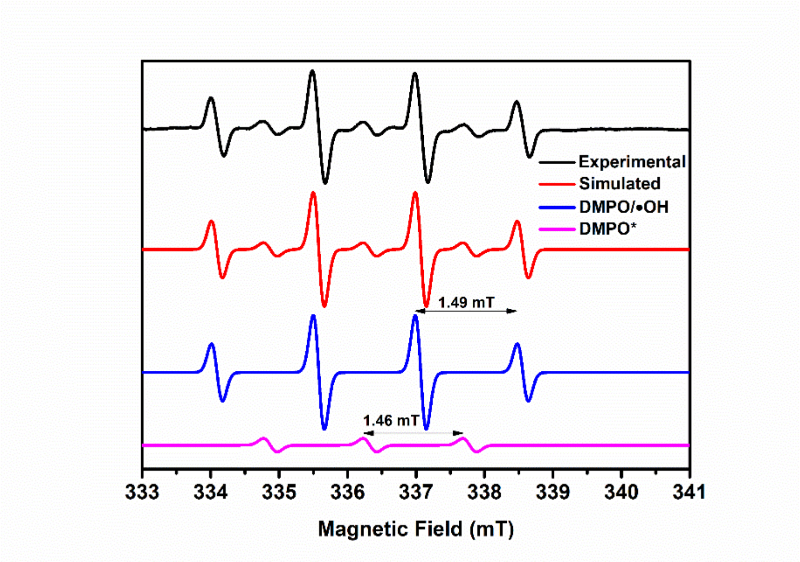

Figure S12. Experimental, simulated, and deconvoluted spectra of paramagnetic species present in the systems. The signal DMPO* are related to DMPO degradation with UV irradiation.

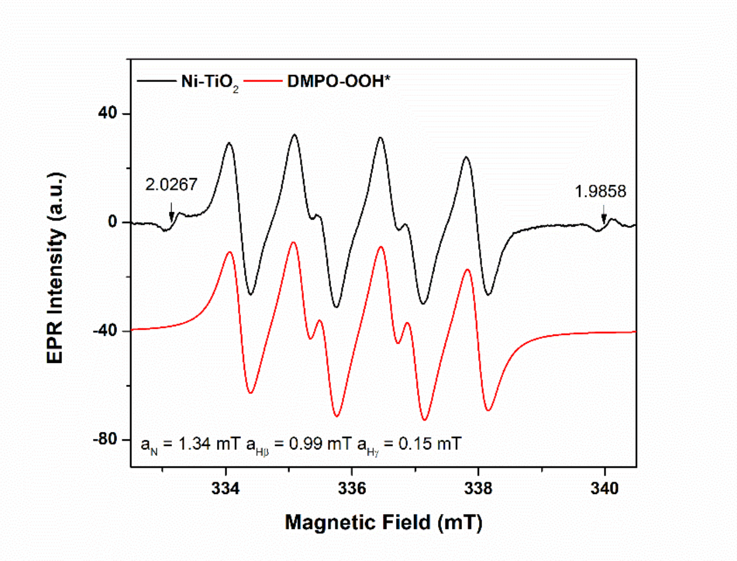

Figure S13. Experimental and simulated spectra of DMPO-OOH paramagnetic species present in the system with Ni-TiO_2 0.5%.

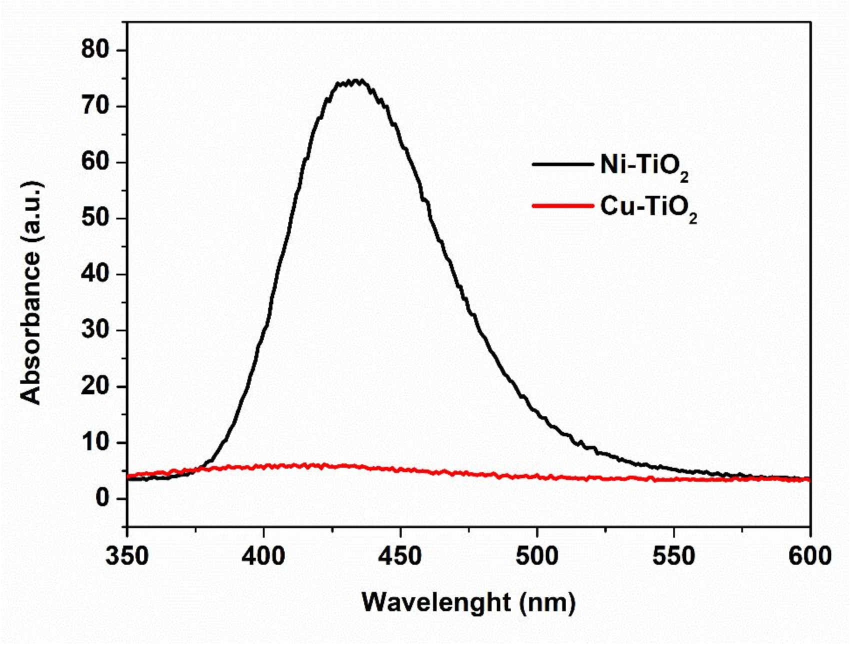

Figure S14. Fluorescence assays using terephthalic acid as probe molecules for hydroxyl radicals. The tests were carried out suing aliquots of reactions without H_2O_2.

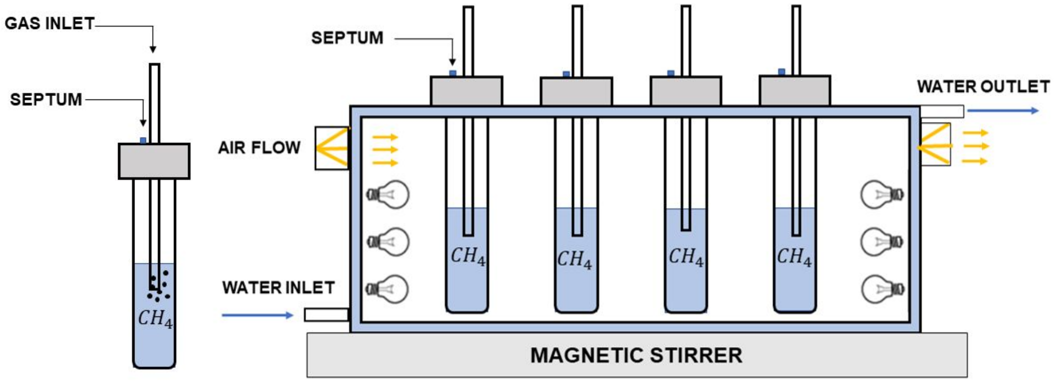

Figure S15. Experimental setup for the methane photo-oxidation reactions. On the left is the schematic of the quartz reactor (140 mL).

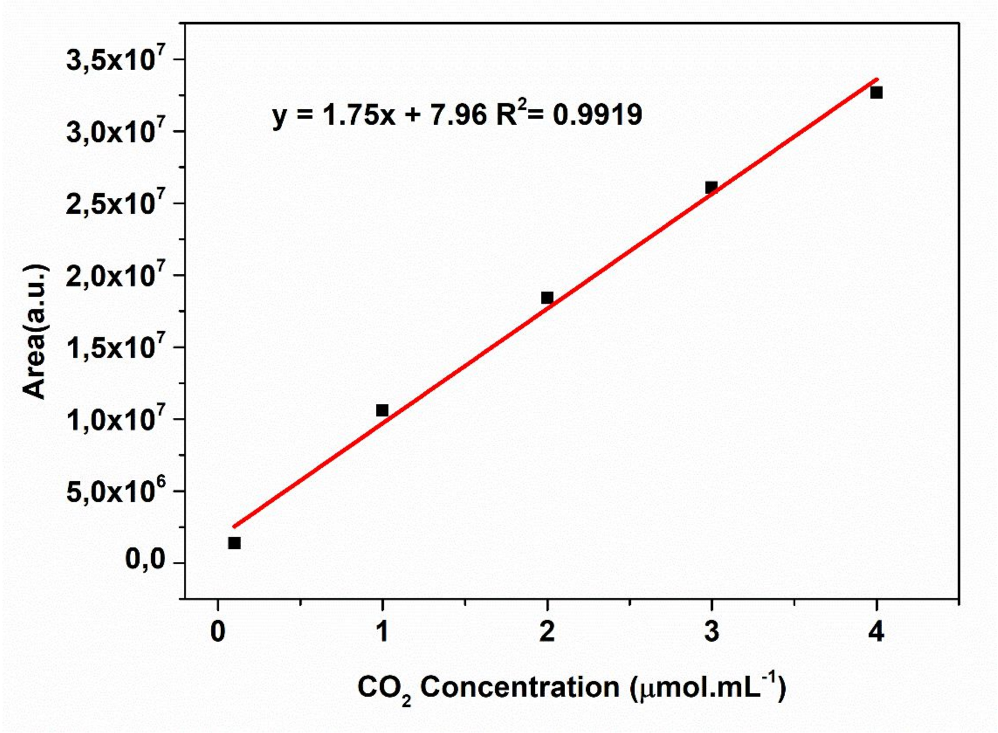

Figure S16. Calibration curve of CO_2 calculated by GC-TCD.

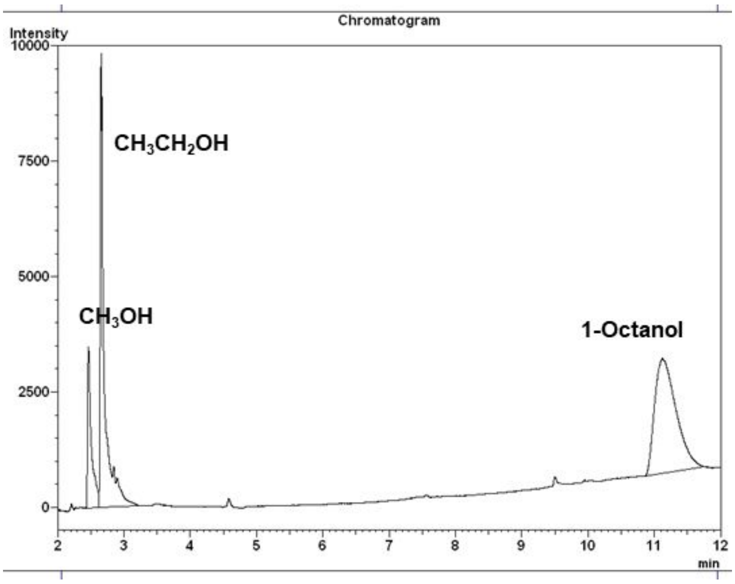

Figure S17. Chromatogram of equimolar quantities of methanol and ethanol with 1-Octanol (1,5 mM) as external standard.

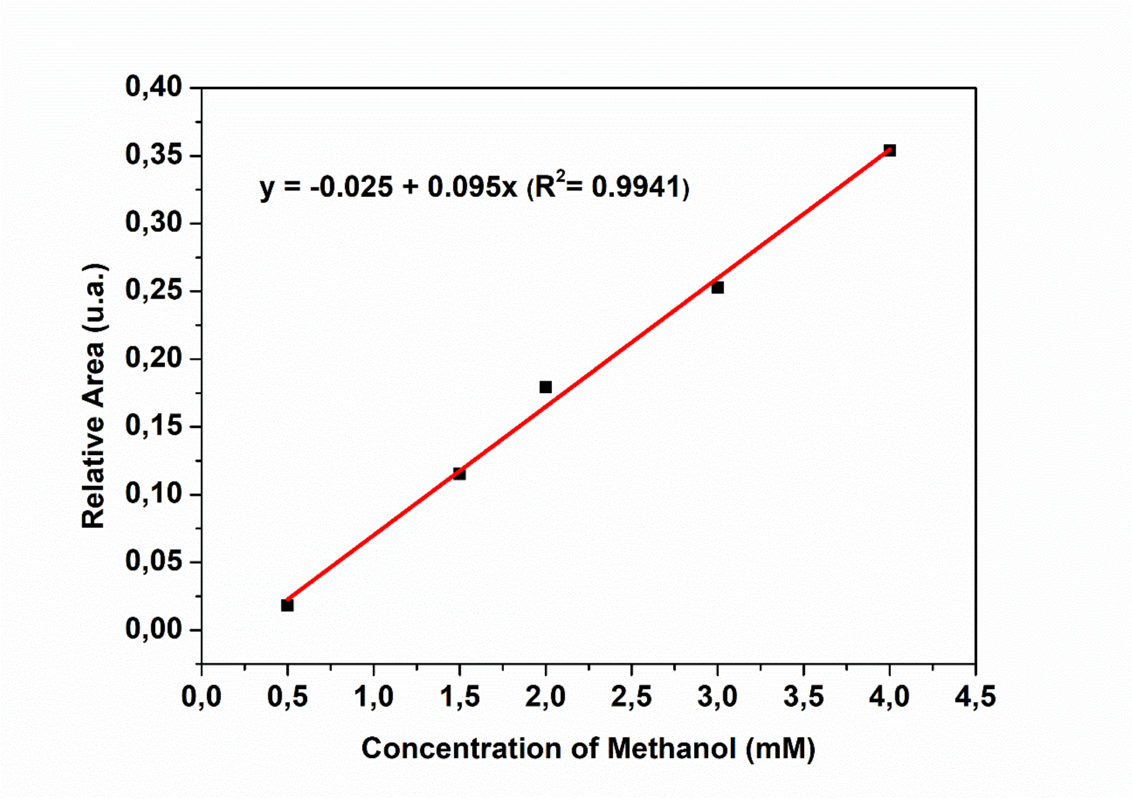

Figure S18. Calibration curve of methanol calculated by GC-FID using 1-Octanol as external standard.

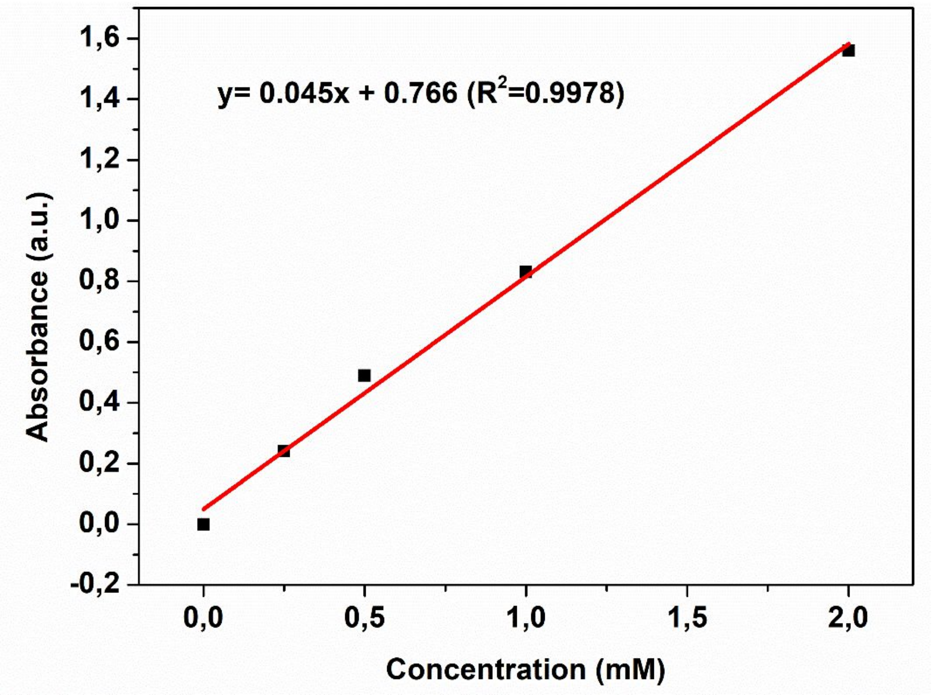

Fig S19. Calibration curve for formaldehyde quantification by colorimetric method.

TABLES

Table S1. FHWM and mean crystallite size (D-Scherrer) for TiO_2 calculated by the Scherrer equation.

Photocatalyst	2θ (deg)	FWHM (rad)	D-Scherrer (nm)*
TiO_2	25,38785	0,005455	26,0546
Ni-TiO_2	25,38641	0,005672	25,0573
Ni-TiO_2 1%	25,45948	0,006188	22,9734
Ni-TiO_2 2%	25,38647	0,004984	28,2151
Cu-TiO_2	25,39769	0,005822	24,411
Co-TiO_2	25,41074	0,005427	26,1883
Ag-TiO_2	25,36978	0,005366	26,4877
Pd-TiO_2	25,28941	0,005398	26,3261

*Crystallite size for TiO2 calculated from the (101) plane corresponding to the most intense peak.

Table S2. Metal content (by AAS and ICP), and specific surface area for the synthesized materials.

Photocatalyst	Metal (wt%)	S_{BET} ($m^2.g^{-1}$)
TiO_2	-	7,8
Ag 0.5%	0,27	10,1
Pd 0.5%	0.48	10,5
Cu 0.5%	0,50	8,7
Co 0.5%	0,50	8,4
Ni 0.5%	0,37	
Ni 1%	1,09	8,0
Ni 2%	2,19	7,0

Table S3. Methane photo-oxidation results of the catalysts synthesized in this work.

Photocatalyst	Production (µmol.g^{-1})			
	CH_3OH	CH_3OOH	CO_2	C_2 Products
TiO_2	200	0	50	140
Co-TiO_2	10	0	0	100
Cu-TiO_2	10	0	0	100
Ag-TiO_2	60	120	170	80
Pd-TiO_2	430	20	185	120
Ni-TiO_2	780	0	0	80
Ni-TiO_2 1%	730	-	-	-
Ni-TiO_2 2%	420	-	-	-
Ni-TiO_2 0.5%*	1561	-	-	-

*Reaction at 16h

Table S4. Comparison of CH_3OH quantification using NMR and GC methods.

Reaction	NMR (µmol.g^{-1})	GC (µmol.g^{-1})
TiO_2 (4h)	200.2	192
Ni-TiO_2 0.5% (4h)	1599.9	1520
Ni-TiO_2 0.5% (3h)	1292.6	1160
Ni-TiO_2 0.5% (2h)	776.9	879.1
Ni-TiO_2 0.5% (4h)*	1116.2	1052.5
Ni-TiO_2 0.5% (2h)*	879.1	915.7
Ni-TiO_2 1% (4h)	730	787.6
Ni-TiO_2 2% (4h)	420	492.4
Pd-TiO_2	430	564.6

* 0.8 mM H_2O_2

Table S5. Comparison of different results in the literature for CH_4 oxidation to CH_3OH.

				Production (µmol.g^{-1})		
**Catalyst**	**Oxidant**	**Pressure**	**Time (h)**	**CH_3OH**	**Total Oxygenates**	**Reference**
Ni-TiO_2 0.5%	H_2O_2 (200 µmol)	CH_4 (1 bar)	4	1600	1770	This Work
FeO_x/TiO_2 0.33 wt%	H_2O_2 (80 µmol)	CH_4 (1 bar)	3	1056	1156	1
NiCN	H_2O_2 (250 µL)	CH_4 (20 bar)	2	1591	1591	2
Fe_2O_3/WO_3 + 1 mM $FeCl_3$	H_2O_2 (0.06 M)	CH_4 (20 bar)	2	5600	30600	3
Bi_2O_3	H_2O_2 (80 µmol)	CH_4 (1 bar)	4	2904	4636	4
Pd_1/DT	H_2O_2 (200 µL)	CH_4 (20 bar)	4	175	186,2	5
BiOCl-O_v	H_2O_2 (500 µmol)	CH_4/N_2 (1:9) (1 bar)	1	180,75	225,75	6
Au_1/WO_3	H_2O_2 (200 µL)	CH_4 (20 bar)	1	589	775	7
0.1%-FeO_x/Z-450	H_2O_2 (5 mM)	CH_4 (30 bar)	3	1680	12324	8
Cu-0.5/PCN	H_2O	CH_4/N_2 (1:9) (1 bar)	1	24.5	130.5	9
Au0.30/ZnO	O_2 (5 bar)	CH_4 (15 bar)	2	1996	2403	10

RCN-5	O_2 (1 bar)	CH_4 (20 bar)	3	75	925	11
1.98%FeOOH/m-WO_3	H_2O_2 (1.5 mM)	CH_4/N_2 (1:9) (1 bar)	4	844.8	844.8	12
Au1/BP	O_2 (3 bar)	CH_4 (30 bar)	2	113.5	113.5	13
RhB/TiO_2	H_2O_2 (50 µL)	CH_4 (20 bar)	4	572	572	14
1.0% Cu-SAs/C_3N_4*	H_2O_2 (5 mL)	CH_4 (30 bar)	5	397	3500	15
2.7% FeN_4/GN*	H_2O_2 (5 mL)	CH_4 (20 bar)	10	-	2300	16

REFERENCES

(1) Xie, J.; Jin, R.; Li, A.; Bi, Y.; Ruan, Q.; Deng, Y.; Zhang, Y.; Yao, S.; Sankar, G.; Ma, D. Highly selective oxidation of methane to methanol at ambient conditions by titanium dioxide-supported iron species. *Nature Catalysis* **2018**, *1* (11), 889-896.
(2) Kumar, P.; Antal, P.; Wang, X.; Wang, J.; Trivedi, D.; Fellner, O. F.; Wu, Y. A.; Nemec, I.; Santana, V. T.; Kopp, J.; et al. Partial Thermal Condensation Mediated Synthesis of High-Density Nickel Single Atom Sites on Carbon Nitride for Selective Photooxidation of Methane into Methanol. *Small* **2024**, *20* (15), 2304574. DOI: https://doi.org/10.1002/smll.202304574.
(3) Zhou, W.; Ma, Y.; Zhu, C.; Wang, M.; Zheng, G.; Lu, Y.; Wang, M. Highly Selectively Methane Photooxidation to CH3OH and HCHO over an Integrated Fe2O3/WO3 Heterojunction Greatly Promoted by Iron(III) Chloride. *ACS Catalysis* **2024**, *14* (5), 3606-3615. DOI: 10.1021/acscatal.3c05391.
(4) de Oliveira, J. A.; da Cruz, J. C.; Nascimento, O. R.; Ribeiro, C. Selective CH4 reform to methanol through partial oxidation over Bi2O3 at room temperature and pressure. *Applied Catalysis B: Environmental* **2022**, *318*, 121827. DOI: https://doi.org/10.1016/j.apcatb.2022.121827.
(5) Wu, X.; Zhang, Q.; Li, W.; Qiao, B.; Ma, D.; Wang, S. L. Atomic-Scale Pd on 2D Titania Sheets for Selective Oxidation of Methane to Methanol. *ACS Catalysis* **2021**, *11* (22), 14038-14046. DOI: 10.1021/acscatal.1c03985.
(6) Wang, J.; Li, R.; Zeng, D.; Wang, W.; Zhang, Y.; Zhang, L.; Wang, W. Photocatalytic conversion of methane selectively into oxygenated products in the presence of chloride ions. *Chemical Engineering Journal* **2023**, *452*, 139505. DOI: https://doi.org/10.1016/j.cej.2022.139505.
(7) Zeng, Y.; Tang, Z.; Wu, X.; Huang, A.; Luo, X.; Xu, G. Q.; Zhu, Y.; Wang, S. L. Photocatalytic oxidation of methane to methanol by tungsten trioxide-supported atomic gold at room temperature. *Applied Catalysis B: Environmental* **2022**, *306*, 120919. DOI: https://doi.org/10.1016/j.apcatb.2021.120919.
(8) Hao, Y.; Mao, F.; Shi, J.; Zhao, Y.; Jiang, L.; Sun, N.; Li, L.; Wei, W. Fabrication of FeO3 sites in FeOx/ZnO for efficient and selective photo-catalytic oxidation of methane to liquid oxygenates. *Cell Reports Physical Science* **2022**, *3* (6), 100909. DOI: https://doi.org/10.1016/j.xcrp.2022.100909.
(9) Zhou, Y.; Zhang, L.; Wang, W. Direct functionalization of methane into ethanol over copper modified polymeric carbon nitride via photocatalysis. *Nature communications* **2019**, *10* (1), 1-8.
(10) Zhou, W.; Qiu, X.; Jiang, Y.; Fan, Y.; Wei, S.; Han, D.; Niu, L.; Tang, Z. Highly selective aerobic oxidation of methane to methanol over gold decorated zinc oxide via photocatalysis. *Journal of Materials Chemistry A* **2020**, *8* (26), 13277-13284.

(11) Yang, Z.; Zhang, Q.; Ren, L.; Chen, X.; Wang, D.; Liu, L.; Ye, J. Efficient photocatalytic conversion of CH 4 into ethanol with O 2 over nitrogen vacancy-rich carbon nitride at room temperature. *Chemical Communications* **2021**, *57* (7), 871-874.
(12) Yang, J.; Hao, J.; Wei, J.; Dai, J.; Li, Y. Visible-light-driven selective oxidation of methane to methanol on amorphous FeOOH coupled m-WO3. *Fuel* **2020**, *266*, 117104.
(13) Luo, L.; Luo, J.; Li, H.; Ren, F.; Zhang, Y.; Liu, A.; Li, W.-X.; Zeng, J. Water enables mild oxidation of methane to methanol on gold single-atom catalysts. *Nature communications* **2021**, *12* (1), 1-10.
(14) Wu, X.; Zeng, Y.; Liu, H.; Zhao, J.; Zhang, T.; Wang, S. L. Noble-metal-free dye-sensitized selective oxidation of methane to methanol with green light (550 nm). *Nano Research* **2021**, *14* (12), 4584-4590.
(15) Wu, B.; Yang, R.; Shi, L.; Lin, T.; Yu, X.; Huang, M.; Gong, K.; Sun, F.; Jiang, Z.; Li, S. Cu single-atoms embedded in porous carbon nitride for selective oxidation of methane to oxygenates. *Chemical Communications* **2020**, *56* (93), 14677-14680.
(16) Cui, X.; Li, H.; Wang, Y.; Hu, Y.; Hua, L.; Li, H.; Han, X.; Liu, Q.; Yang, F.; He, L. Room-temperature methane conversion by graphene-confined single iron atoms. *Chem* **2018**, *4* (8), 1902-1910.
